# Supplementary figures and images for: A systematic review and meta-analysis of factors related to first line drugs refractoriness in patients with juvenile myoclonic epilepsy (JME)
Source: PLoS One. 2024 Apr 9;19(4):e0300930. doi: 10.1371/journal.pone.0300930 (PMC11003615; doi:10.1371/journal.pone.0300930)

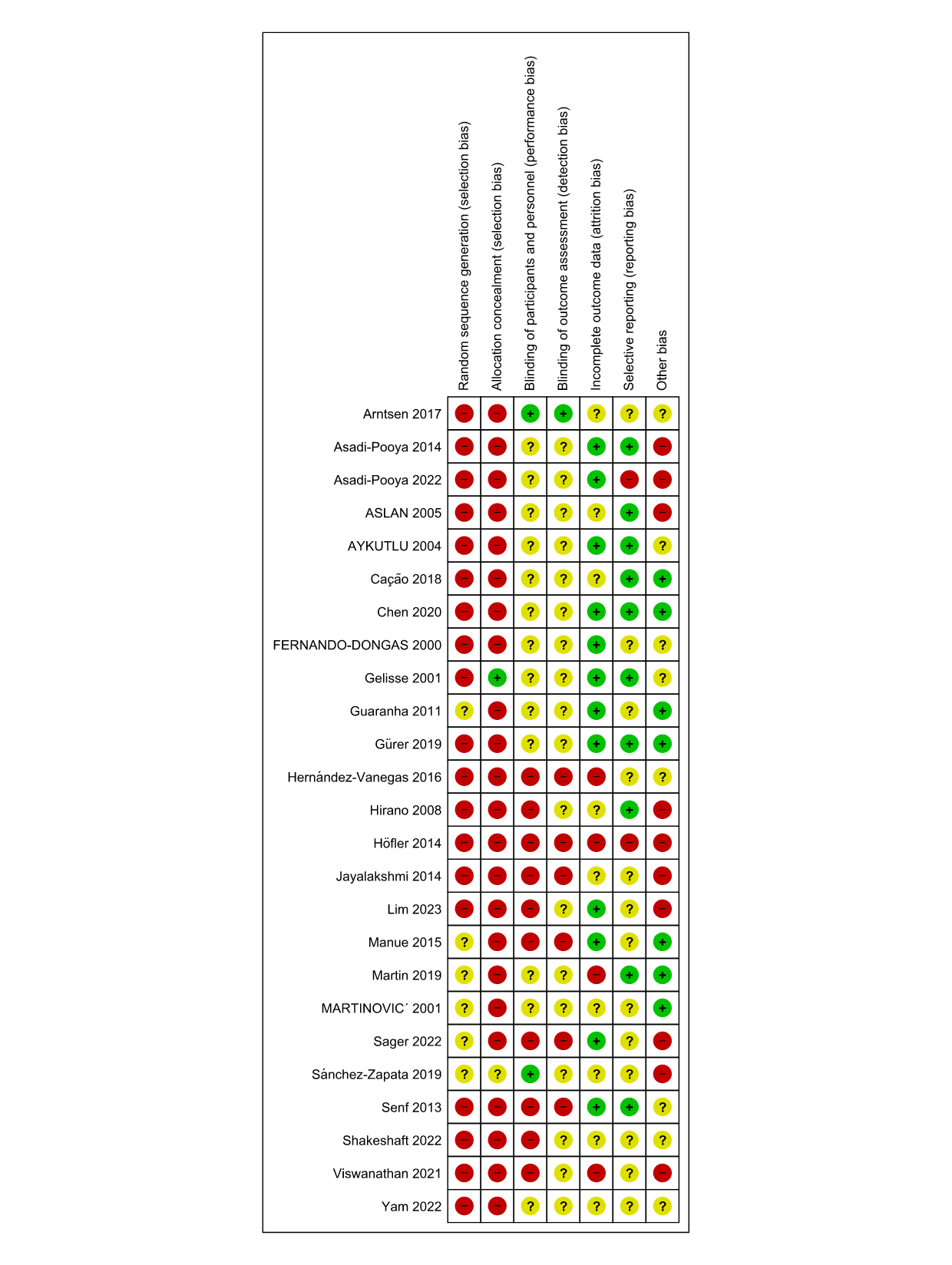

Supplement: S1 Fig — (TIF) [file pone.0300930.s001.tif]
