# Supplementary material for: A systematic review and meta-analysis of factors related to first line drugs refractoriness in patients with juvenile myoclonic epilepsy (JME)
Source: PLoS One. 2024 Apr 9;19(4):e0300930. doi: 10.1371/journal.pone.0300930 (PMC11003615; doi:10.1371/journal.pone.0300930)
Supplement: S1 File — ASM Resistant VS ASM Non-Resistant, done for each outcome to evaluate potential publication biases. (PDF) [file pone.0300930.s002.pdf]

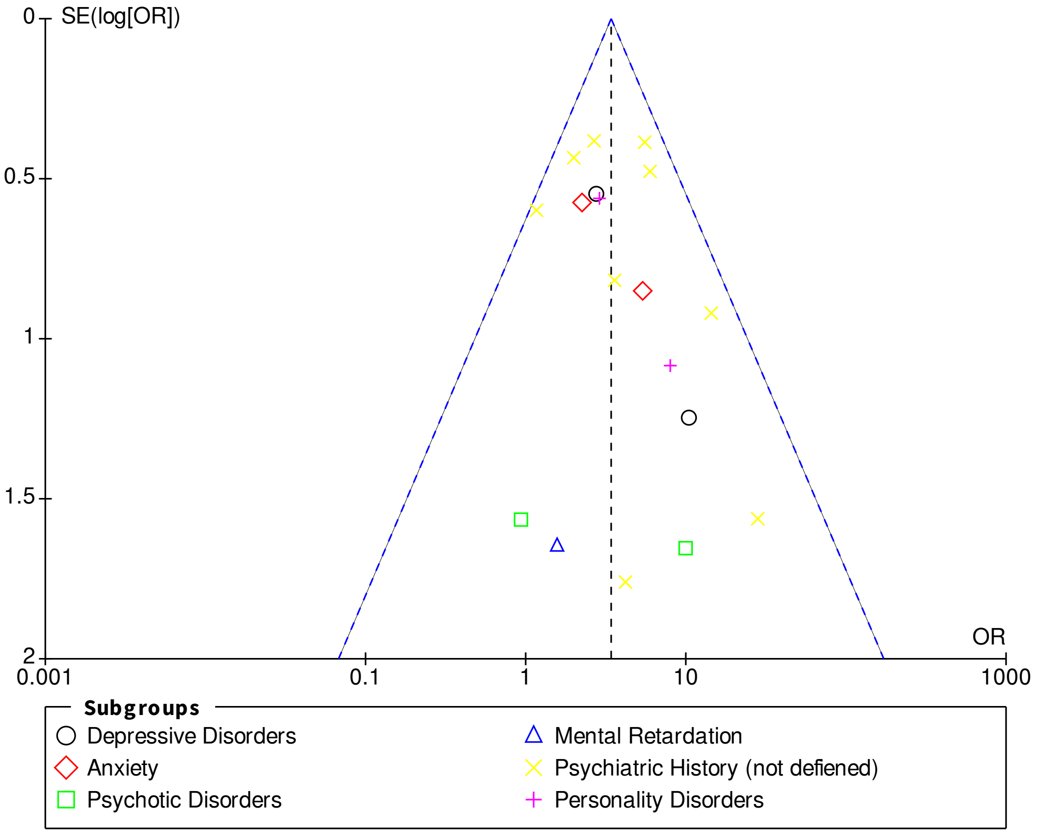

Funnel plot of comparison: 1 AED Resistant VS AED Non-Resistant, outcome: 1.2 Psychiatric disorders.

Figure 3

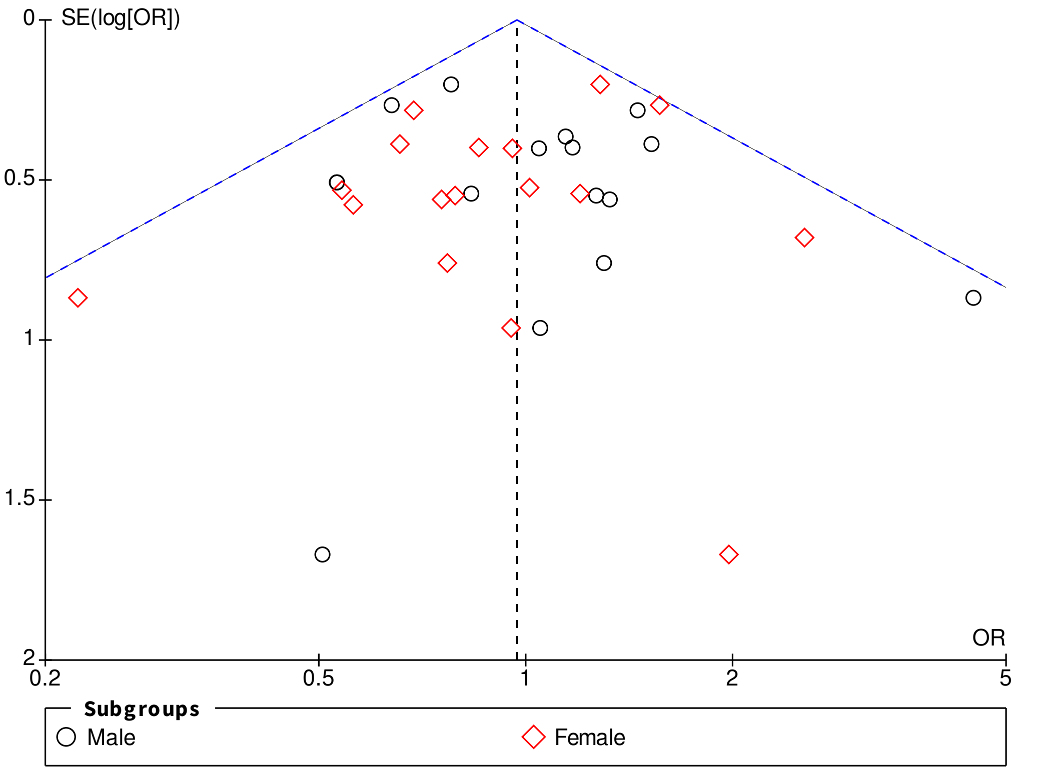

Funnel plot of comparison: 1 AED Resistant VS AED Non-Resistant, outcome: 1.3 Gender.

Figure 4

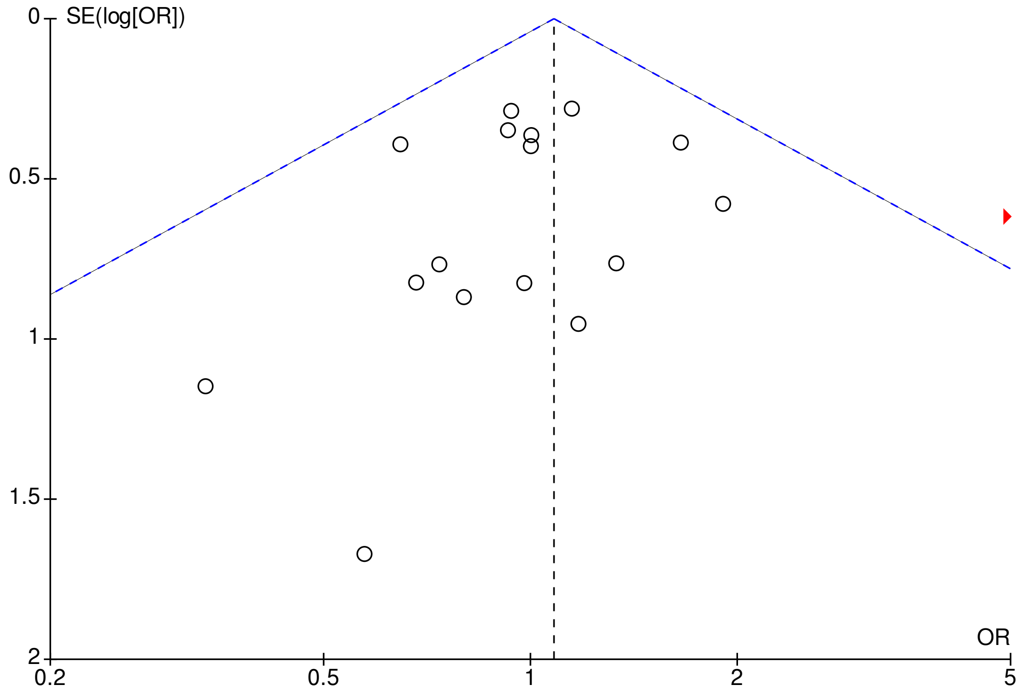

Funnel plot of comparison: 1 AED Resistant VS AED Non-Resistant, outcome: 1.1 Family History.

Figure 5

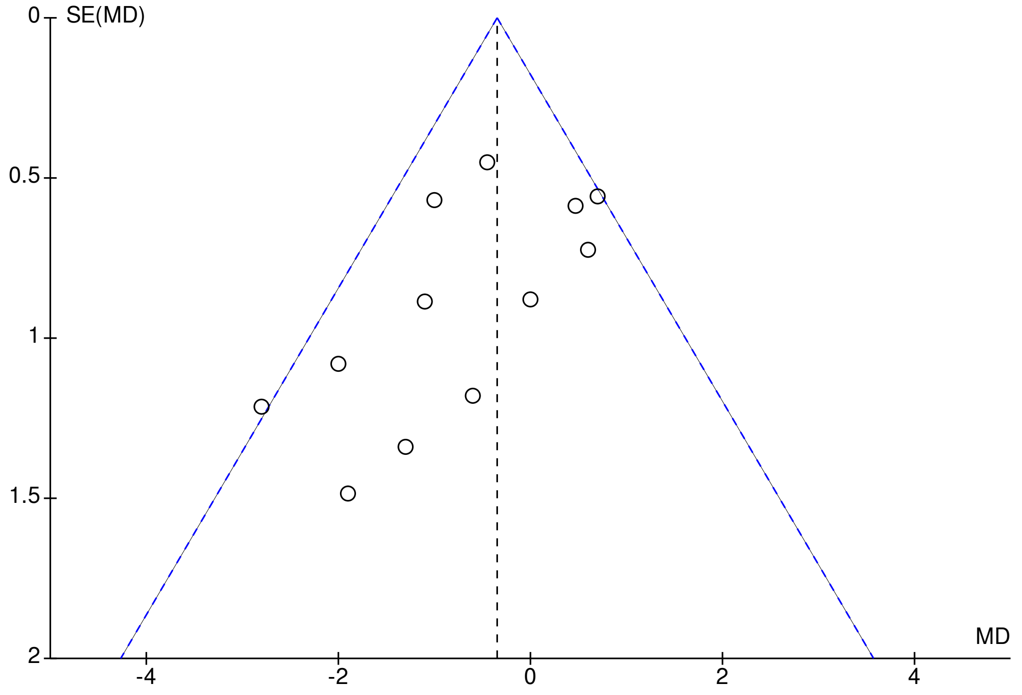

Funnel plot of comparison: 1 AED Resistant VS AED Non-Resistant, outcome: 1.4 Mean Age of Seizures Onset.

Figure 6

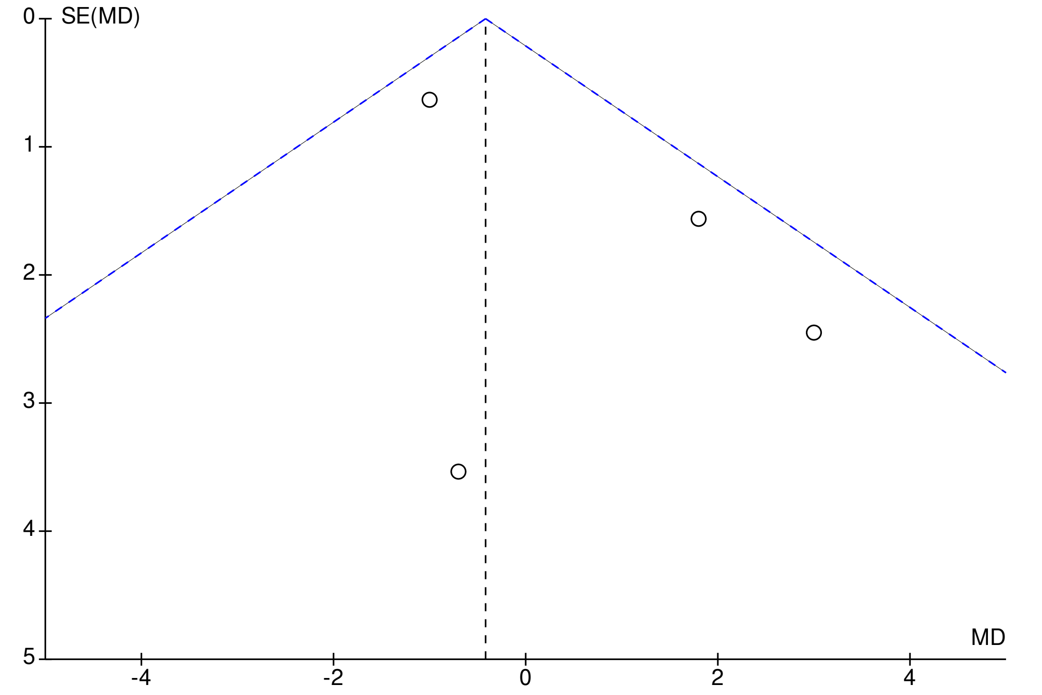

Funnel plot of comparison: 1 AED Resistant VS AED Non-Resistant, outcome: 1.5 Mean Age at Diagnosis.

Figure 7

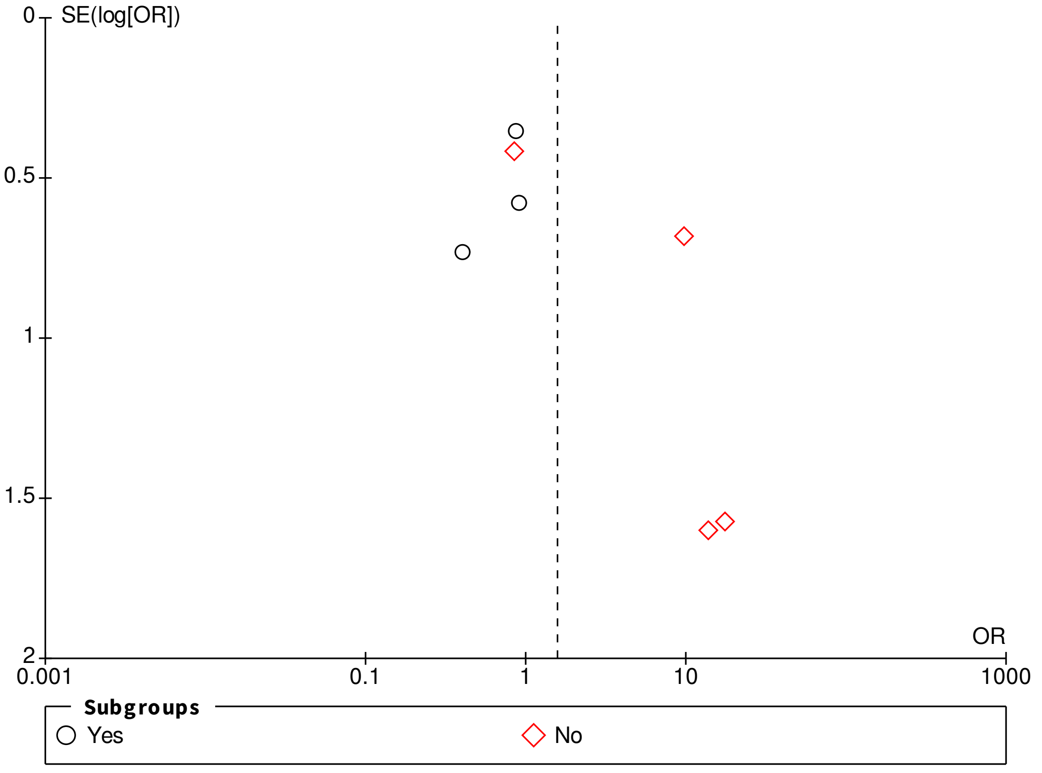

Funnel plot of comparison: 1 AED Resistant VS AED Non-Resistant, outcome: 1.6 Education.

Figure 8

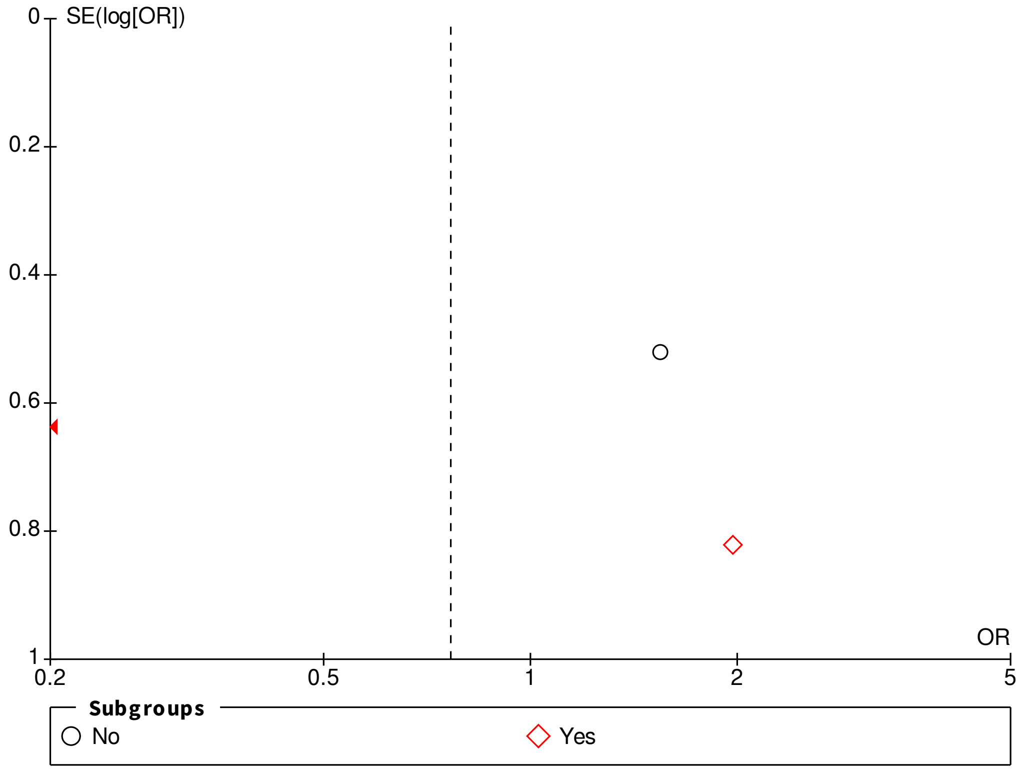

Funnel plot of comparison: 1 AED Resistant VS AED Non-Resistant, outcome: 1.8 low socioeconomic status.

Figure 9

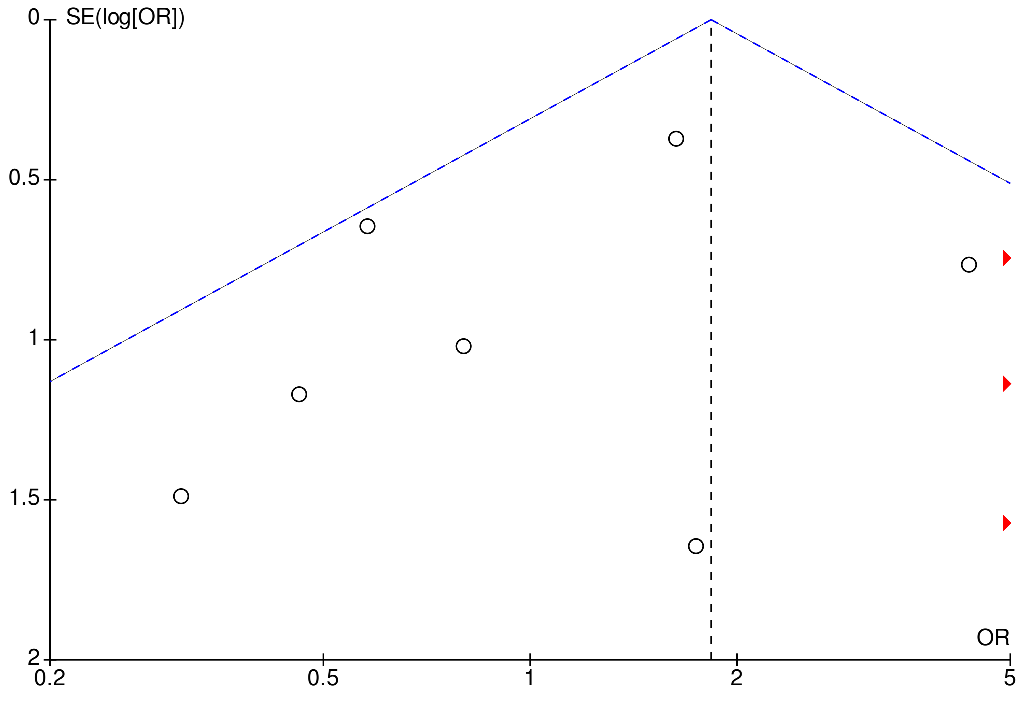

Funnel plot of comparison: 1 AED Resistant VS AED Non-Resistant, outcome: 1.9 Febrile Seizures.

Figure 10

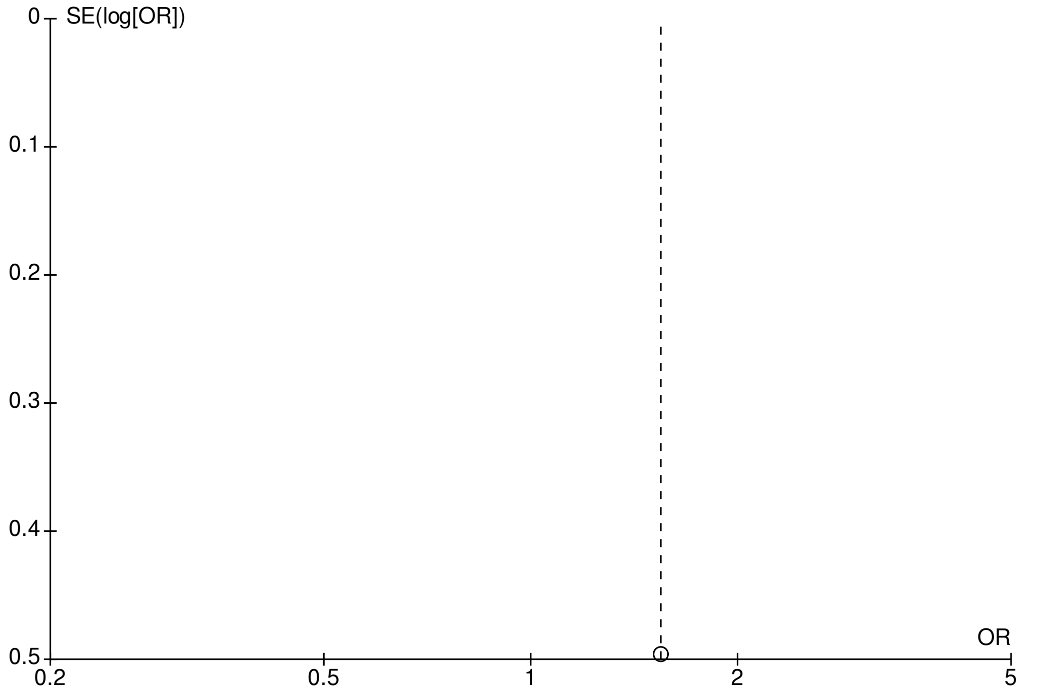

Funnel plot of comparison: 1 AED Resistant VS AED Non-Resistant, outcome: 1.10 Perinatal complications.

Figure 11

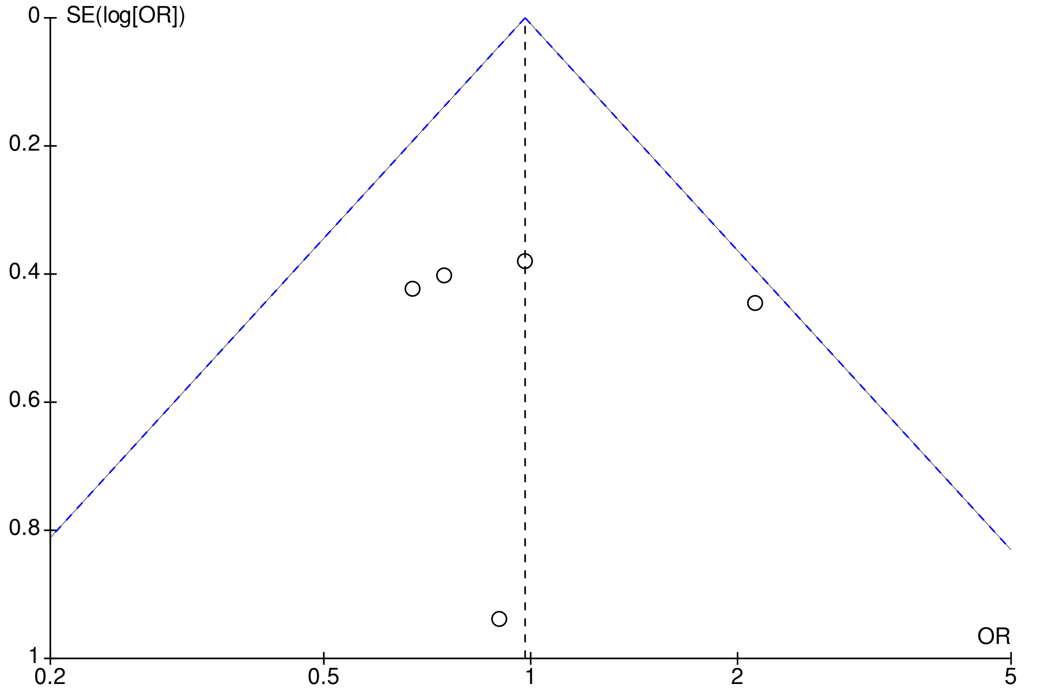

Funnel plot of comparison: 1 AED Resistant VS AED Non-Resistant, outcome: 1.11 Abnormal Neuroimaging.

Figure 12

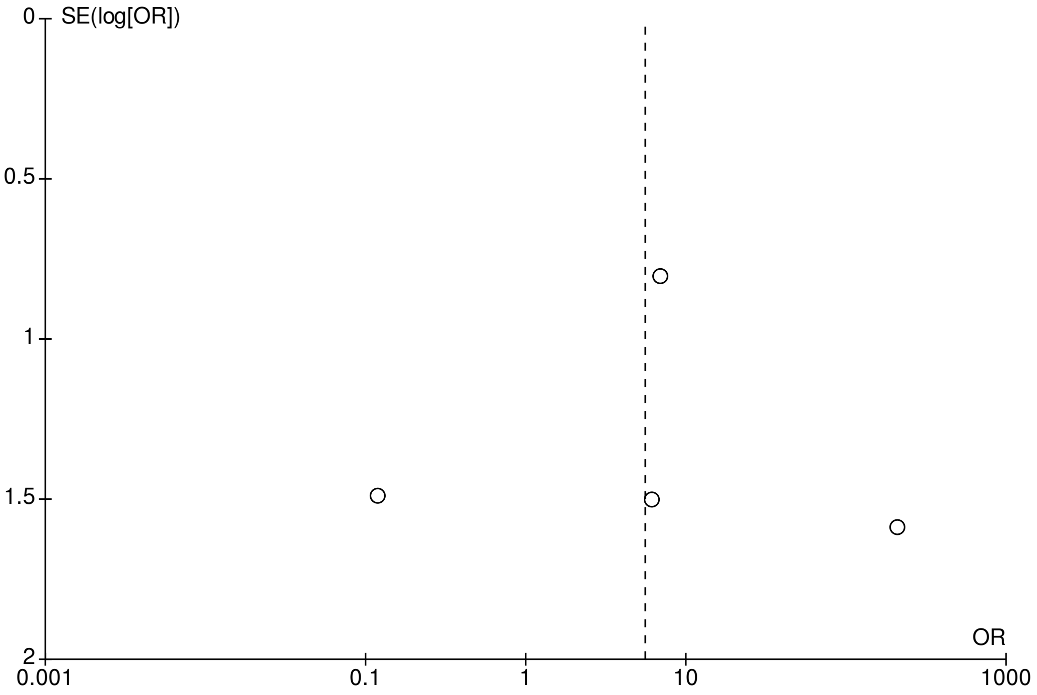

Funnel plot of comparison: 1 AED Resistant VS AED Non-Resistant, outcome: 1.13 Status Epilepticus.

Figure 13

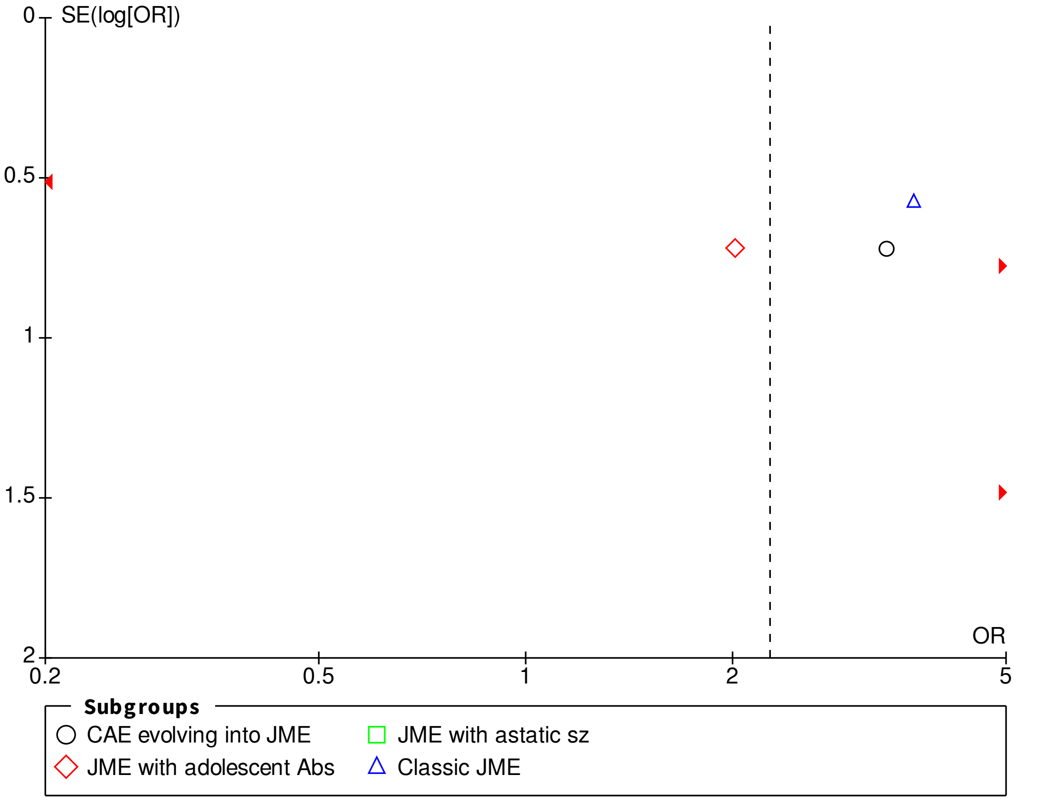

Funnel plot of comparison: 1 AED Resistant VS AED Non-Resistant, outcome: 1.12 Clinical Phenotype.

Figure 14

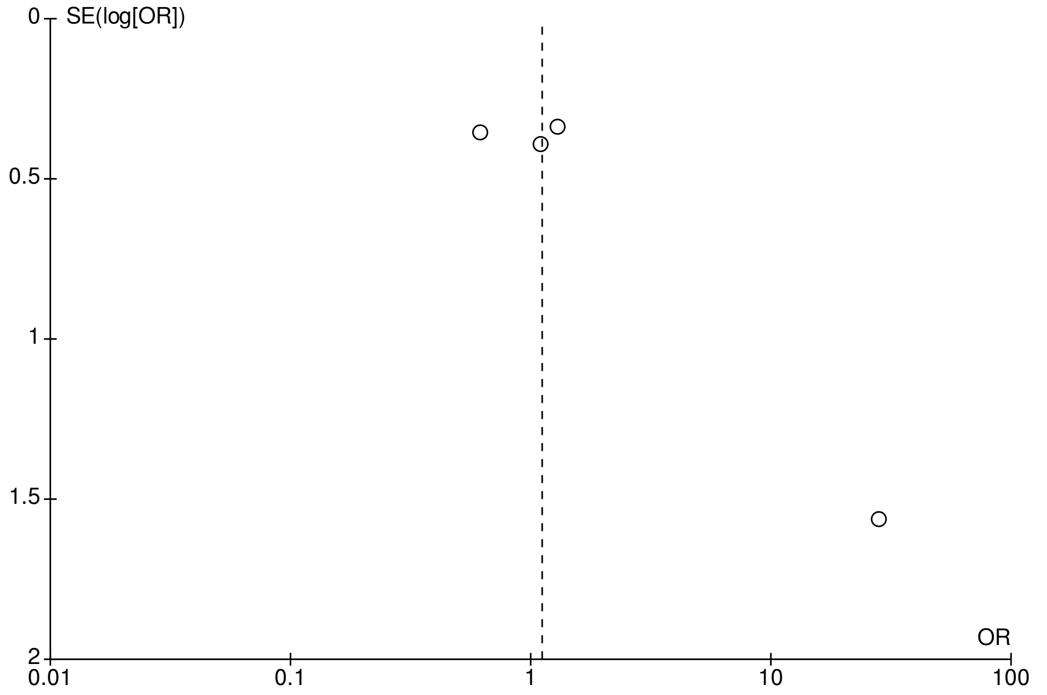

Funnel plot of comparison: 1 AED Resistant VS AED Non-Resistant, outcome: 1.14 Cosanguinity.

Figure 15

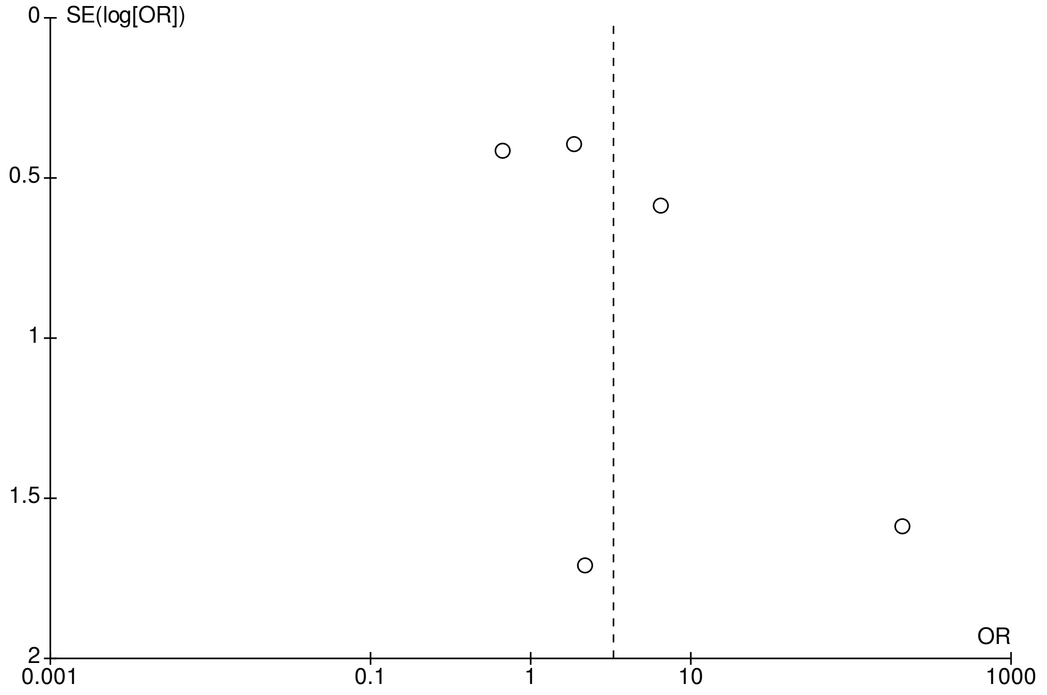

Funnel plot of comparison: 1 AED Resistant VS AED Non-Resistant, outcome: 1.15 Comorbid Conditions.

Figure 16

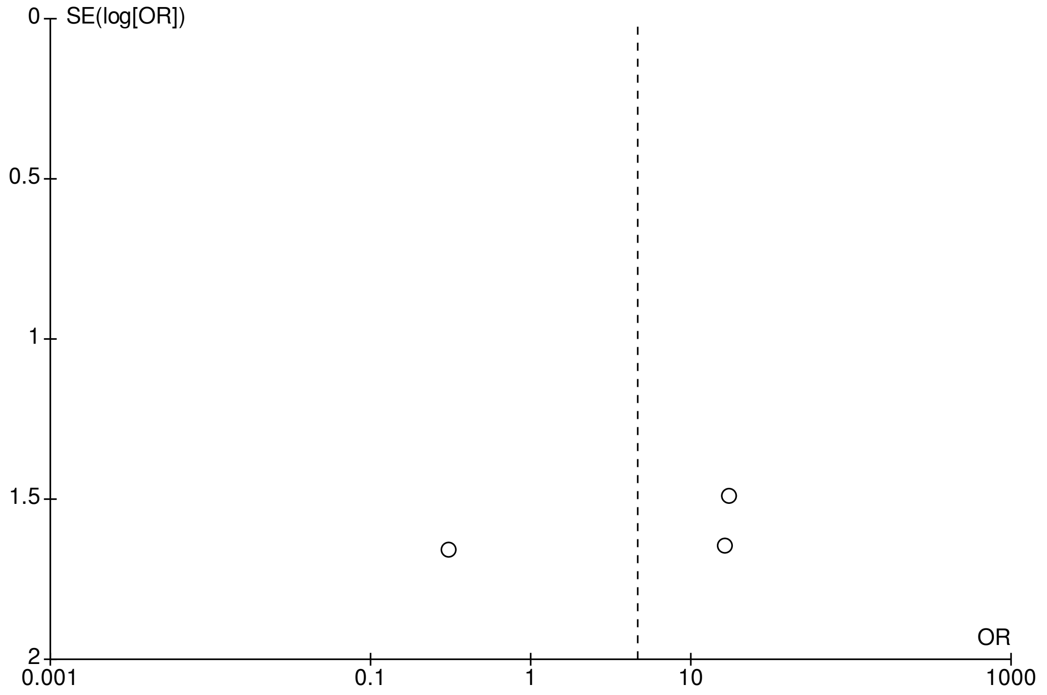

Funnel plot of comparison: 1 AED Resistant VS AED Non-Resistant, outcome: 1.16 Alcohol Consumption.

Figure 17

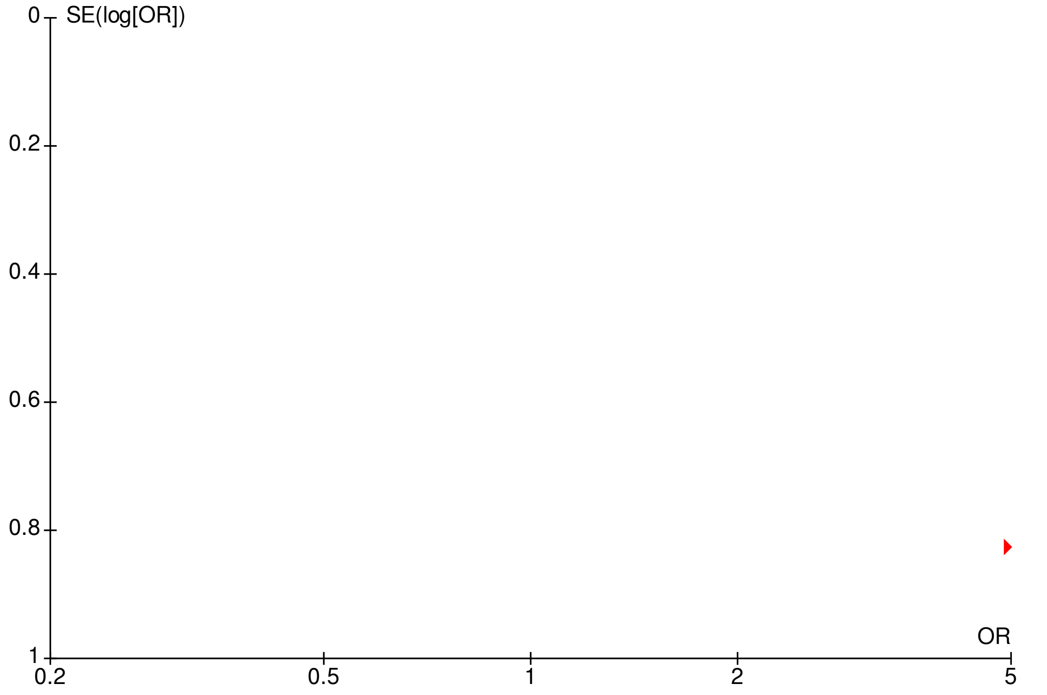

Funnel plot of comparison: 1 AED Resistant VS AED Non-Resistant, outcome: 1.17 Tobacco consumption.

Figure 18

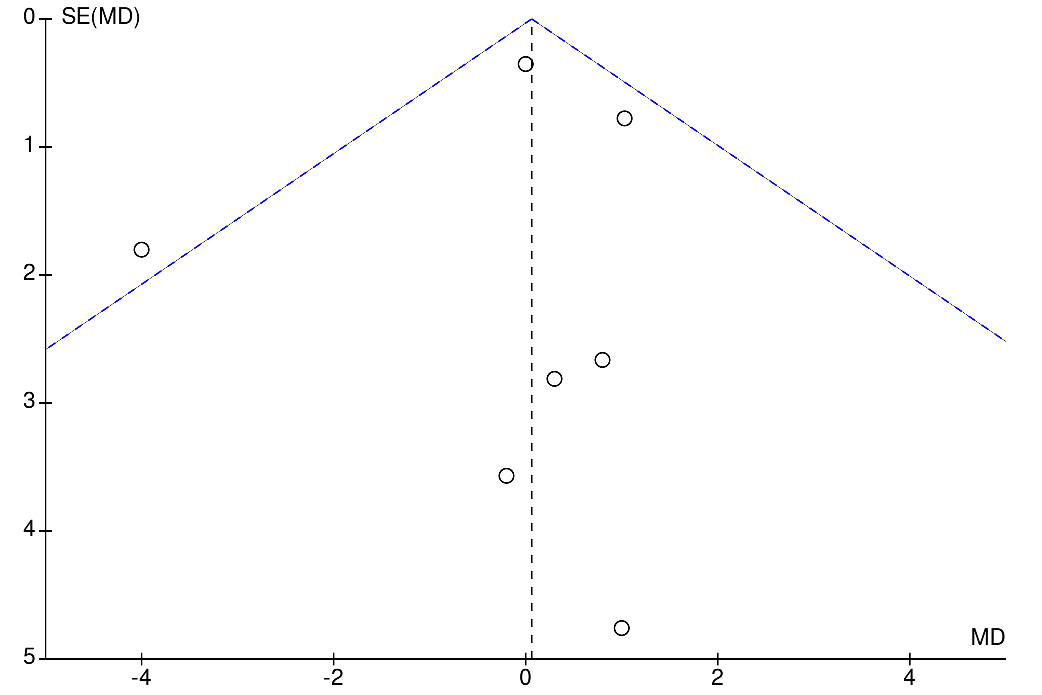

Funnel plot of comparison: 1 AED Resistant VS AED Non-Resistant, outcome: 1.18 Follow Up Time.

Figure 19

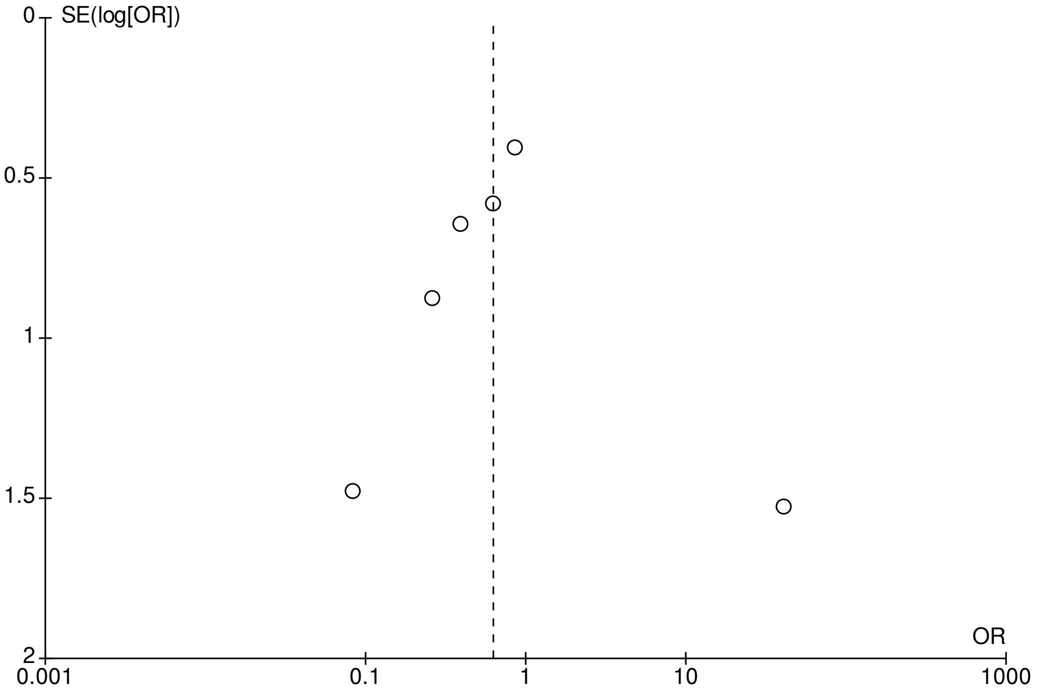

Funnel plot of comparison: 1 AED Resistant VS AED Non-Resistant, outcome: 1.19 Photosensitivity.

Figure 20

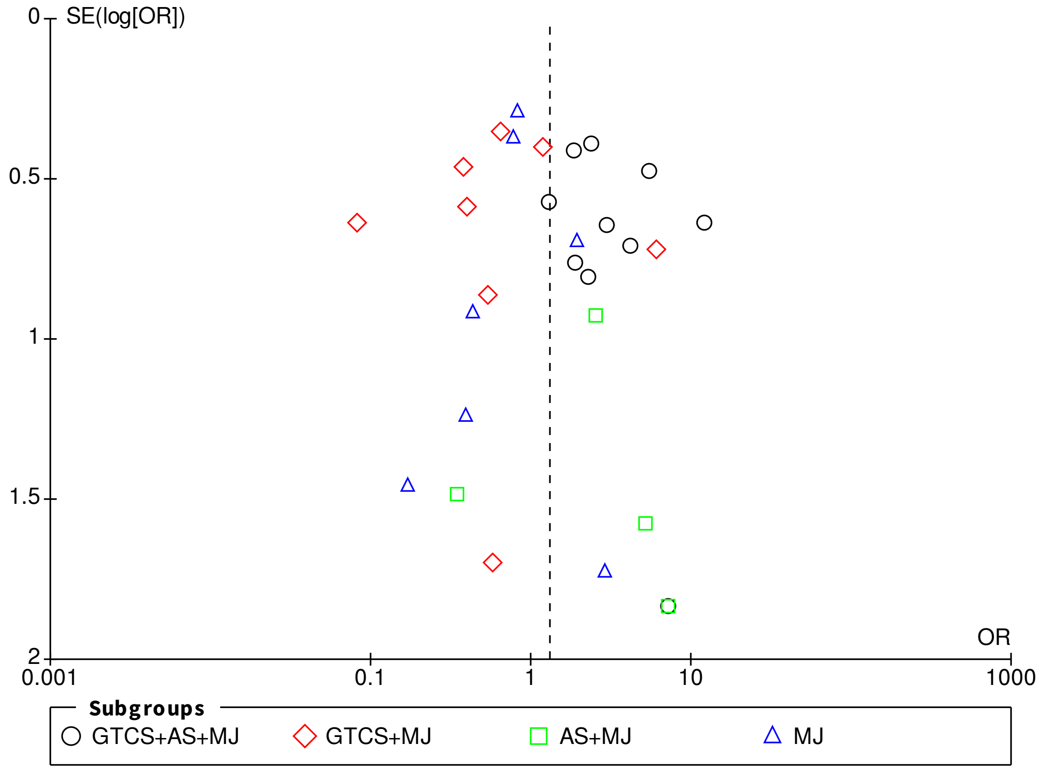

Funnel plot of comparison: 1 AED Resistant VS AED Non-Resistant, outcome: 1.20 Seizure Type.

Figure 21

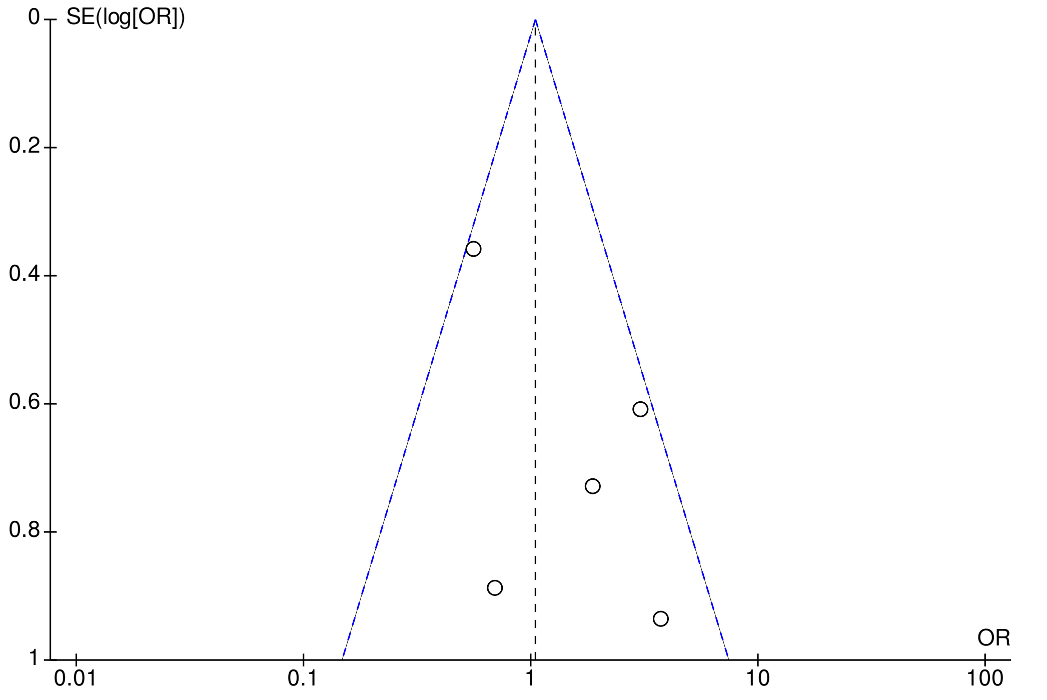

Funnel plot of comparison: 1 AED Resistant VS AED Non-Resistant, outcome: 1.21 Photoparoxysmal Response.

Figure 22

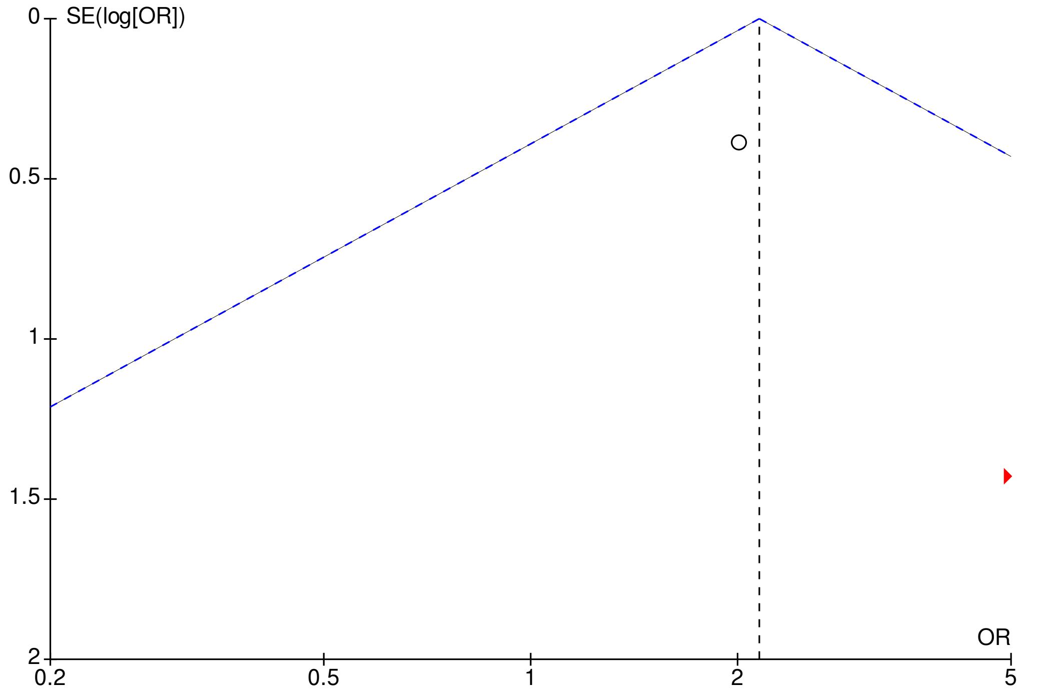

Funnel plot of comparison: 1 AED Resistant VS AED Non-Resistant, outcome: 1.22 Aura.

Figure 23

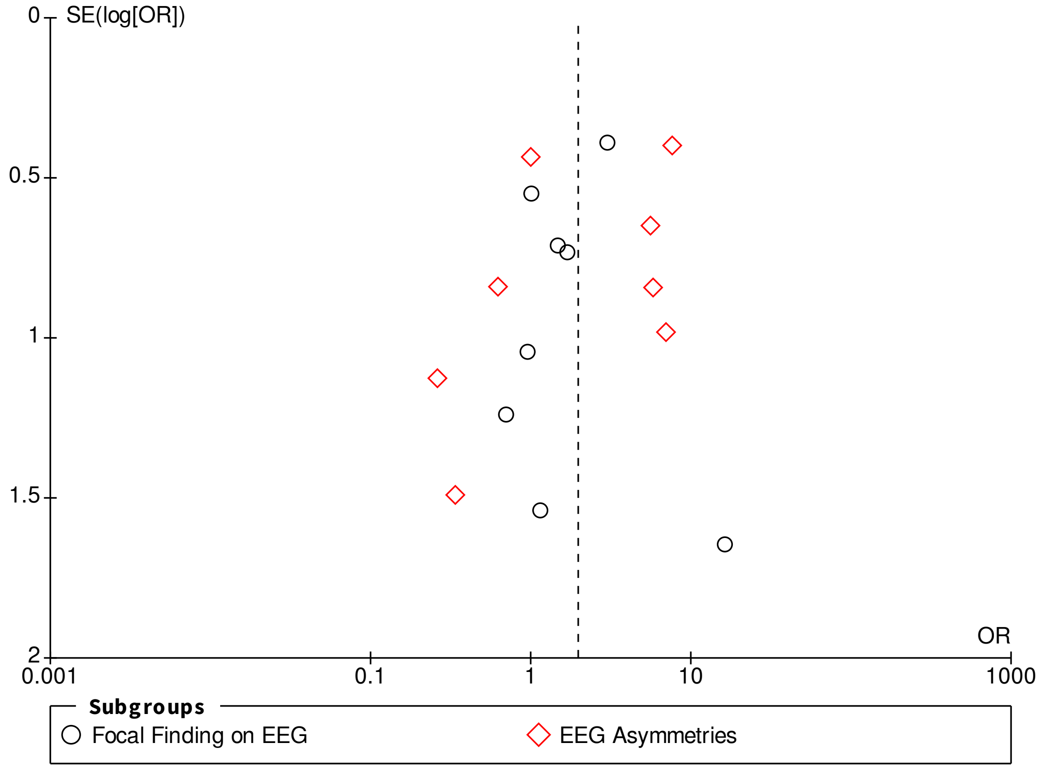

Funnel plot of comparison: 1 AED Resistant VS AED Non-Resistant, outcome: 1.23 Abnormal EEG findings.

Figure 24
