# Supplementary material for: A systematic review and meta-analysis of factors related to first line drugs refractoriness in patients with juvenile myoclonic epilepsy (JME)
Source: PLoS One. 2024 Apr 9;19(4):e0300930. doi: 10.1371/journal.pone.0300930 (PMC11003615; doi:10.1371/journal.pone.0300930)
Supplement: S2 File — ASM Resistant VS ASM Non-Resistant, done for each outcome to depict the interconnection among studies and estimate the association between drug refractoriness and the corresponding risk factor. (PDF) [file pone.0300930.s003.pdf]

Study or Subgroup	Resistant Seizures Events	Resistant Seizures Total	Controlled Seizures Events	Controlled Seizures Total	Weight	Odds Ratio IV, Random, 95% CI	A	B	C	D	E	F	G
**1.20.1 GTCS+AS+MJ**													
AYKUTLU 2004	3	7	20	81	3.3%	2.29 [0.47, 11.10]	−	−	?	?	+	+	?
Chen 2020	4	23	4	40	3.5%	1.89 [0.43, 8.43]	−	−	?	?	+	+	+
Gelisse 2001	15	24	27	116	4.8%	5.49 [2.16, 13.95]	−	+	?	?	+	+	?
Hernández-Vanegas 2016	27	57	15	46	5.1%	1.86 [0.83, 4.17]	−	−	−	−	−	?	?
Hirano 2008	0	1	1	33	1.1%	7.22 [0.20, 262.97]	−	−	−	?	?	+	−
Höfler 2014	7	66	3	109	3.7%	4.19 [1.04, 16.82]	−	−	−	−	−	−	−
Jayalakshmi 2014	14	38	32	163	5.2%	2.39 [1.11, 5.13]	−	−	−	−	?	?	−
Lim 2023	11	25	5	24	4.0%	2.99 [0.84, 10.55]	−	−	−	?	+	?	−
MARTINOVIC´ 2001	8	22	11	36	4.3%	1.30 [0.42, 3.99]	?	−	?	?	?	?	+
Sager 2022	15	21	7	41	4.0%	12.14 [3.49, 42.31]	?	−	−	−	+	?	−
**Subtotal (95% CI)**		**284**		**689**	**39.1%**	**2.96 [1.96, 4.46]**							
Total events	104		125										
Heterogeneity: $Tau^2 = 0.08$; $Chi^2 = 11.17$, df = 9 (P = 0.26); $I^2 = 19\%$													
Test for overall effect: Z = 5.18 (P < 0.00001)													
**1.20.2 GTCS+MJ**													
Arntsen 2017	15	19	8	21	3.6%	6.09 [1.49, 25.00]	−	−	+	+	?	?	?
Chen 2020	20	23	37	40	3.1%	0.54 [0.10, 2.93]	−	−	?	?	+	+	+
Gelisse 2001	9	24	71	116	4.9%	0.38 [0.15, 0.94]	−	+	?	?	+	+	?
Hernández-Vanegas 2016	26	57	19	46	5.2%	1.19 [0.54, 2.61]	−	−	−	−	−	?	?
Hirano 2008	1	1	28	33	1.2%	0.58 [0.02, 16.15]	−	−	−	?	?	+	−
Höfler 2014	16	66	36	109	5.4%	0.65 [0.33, 1.29]	−	−	−	−	−	−	−
Lim 2023	10	25	15	24	4.3%	0.40 [0.13, 1.26]	−	−	−	?	+	?	−
MARTINOVIC´ 2001	22	22	36	36		Not estimable	?	−	?	?	?	?	+
Sager 2022	6	21	34	41	4.0%	0.08 [0.02, 0.29]	?	−	−	−	+	?	−
**Subtotal (95% CI)**		**258**		**466**	**31.7%**	**0.60 [0.28, 1.29]**							
Total events	125		284										
Heterogeneity: $Tau^2 = 0.77$; $Chi^2 = 24.50$, df = 7 (P = 0.0009); $I^2 = 71\%$													
Test for overall effect: Z = 1.31 (P = 0.19)													
**1.20.3 AS+MJ**													
Gelisse 2001	0	24	6	116	1.5%	0.35 [0.02, 6.37]	−	+	?	?	+	+	?
Hirano 2008	0	1	1	33	1.1%	7.22 [0.20, 262.97]	−	−	−	?	?	+	−
Höfler 2014	3	66	2	109	2.8%	2.55 [0.41, 15.66]	−	−	−	−	−	−	−
Lim 2023	2	25	0	24	1.4%	5.21 [0.24, 114.41]	−	−	−	?	+	?	−
**Subtotal (95% CI)**		**116**		**282**	**6.8%**	**2.23 [0.62, 8.09]**							
Total events	5		9										
Heterogeneity: $Tau^2 = 0.00$; $Chi^2 = 2.29$, df = 3 (P = 0.51); $I^2 = 0\%$													
Test for overall effect: Z = 1.22 (P = 0.22)													
**1.20.4 MJ**													
Chen 2020	5	23	5	40	3.8%	1.94 [0.50, 7.60]	−	−	?	?	+	+	+
Gelisse 2001	0	24	12	116	1.6%	0.17 [0.01, 2.98]	−	+	?	?	+	+	?
Gürer 2019	29	83	52	132	5.7%	0.83 [0.47, 1.46]	−	−	?	?	+	+	+
Hernández-Vanegas 2016	1	57	2	46	2.0%	0.39 [0.03, 4.47]	−	−	−	−	−	?	?
Hirano 2008	0	1	3	33	1.2%	2.90 [0.10, 85.86]	−	−	−	?	?	+	−
Höfler 2014	14	66	28	109	5.3%	0.78 [0.38, 1.62]	−	−	−	−	−	−	−
Lim 2023	2	25	4	24	2.9%	0.43 [0.07, 2.63]	−	−	−	?	+	?	−
**Subtotal (95% CI)**		**279**		**500**	**22.4%**	**0.82 [0.55, 1.22]**							
Total events	51		106										
Heterogeneity: $Tau^2 = 0.00$; $Chi^2 = 4.08$, df = 6 (P = 0.67); $I^2 = 0\%$													
Test for overall effect: Z = 0.97 (P = 0.33)													
**Total (95% CI)**		**937**		**1937**	**100.0%**	**1.32 [0.87, 1.99]**							
Total events	285		524										
Heterogeneity: $Tau^2 = 0.69$; $Chi^2 = 82.06$, df = 28 (P < 0.00001); $I^2 = 66\%$													
Test for overall effect: Z = 1.32 (P = 0.19)													
Test for subgroup differences: $Chi^2 = 24.83$, df = 3 (P < 0.0001), $I^2 = 87.9\%$													

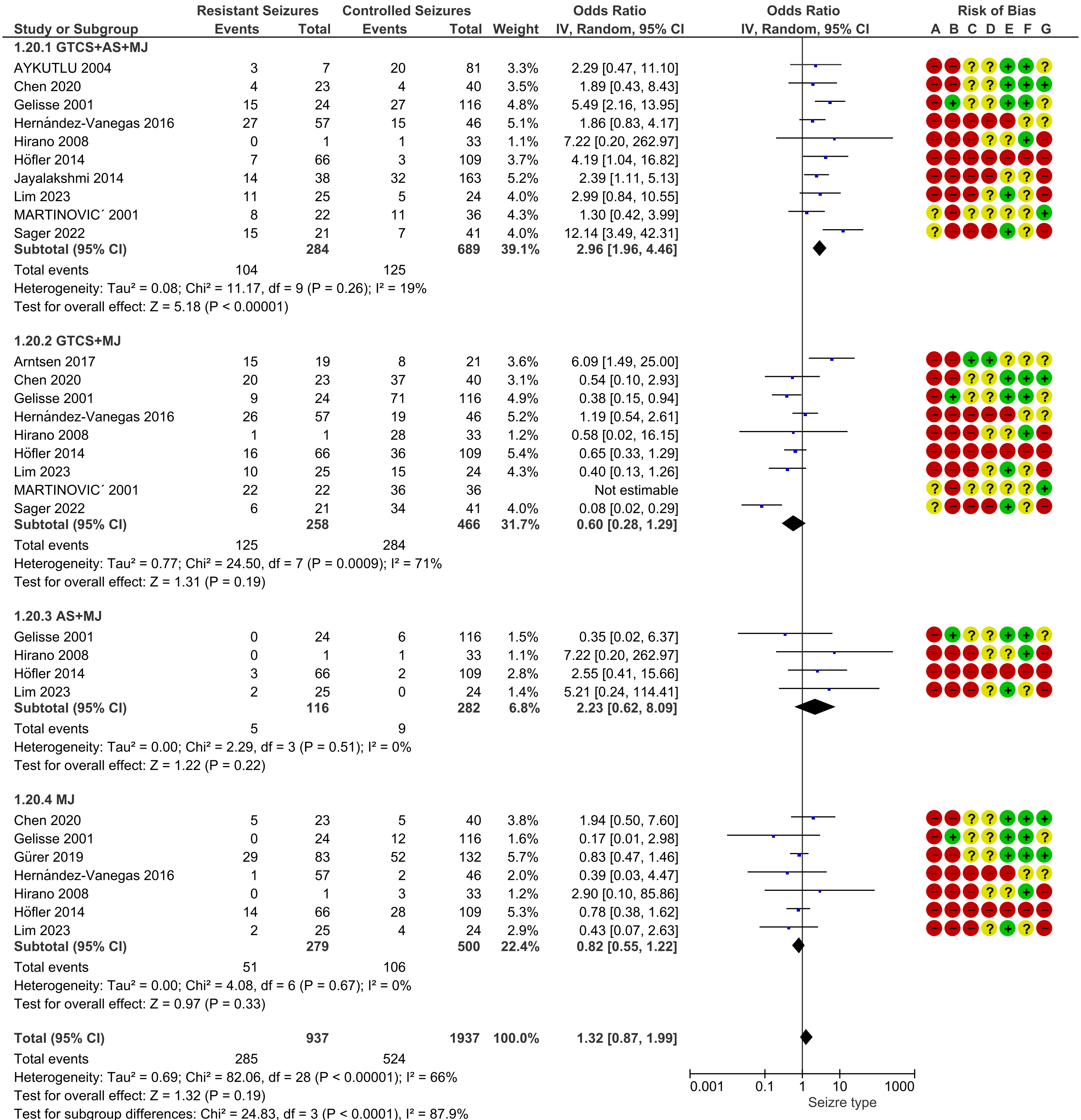

Risk of bias legend

(**A**) Random sequence generation (selection bias)
(**B**) Allocation concealment (selection bias)
(**C**) Blinding of participants and personnel (performance bias)
(**D**) Blinding of outcome assessment (detection bias)
(**E**) Incomplete outcome data (attrition bias)
(**F**) Selective reporting (reporting bias)
(**G**) Other bias

Study or Subgroup	AEDs resistant Events	AEDs resistant Total	AEDs non resistant Events	AEDs non resistant Total	Weight	Odds Ratio IV, Random, 95% CI	A	B	C	D	E	F	G
**1.23.1 Focal Finding on EEG**													
Arntsen 2017	6	19	5	21	7.2%	1.48 [0.37, 5.96]	−	−	+	+	?	?	?
Asadi-Pooya 2014	1	48	2	68	3.6%	0.70 [0.06, 7.97]	−	−		?	+	+	−
AYKUTLU 2004	0	7	4	81	2.6%	1.15 [0.06, 23.44]	−	−	?	?	+	+	?
Chen 2020	15	23	26	40	9.0%	1.01 [0.34, 2.96]	−	−	?	?	+	+	+
Jayalakshmi 2014	15	38	29	163	10.9%	3.01 [1.40, 6.47]	−	−	−	−	?	?	−
Lim 2023	2	25	2	24	4.6%	0.96 [0.12, 7.40]	−	−	−	?	+	?	−
Manue 2015	1	30	0	160	2.3%	16.32 [0.65, 410.39]	?	−	−	−	+		+
Sager 2022	4	21	5	41	7.0%	1.69 [0.40, 7.12]	?	−	−	−	+	?	−
**Subtotal (95% CI)**		**211**		**598**	**47.1%**	**1.85 [1.13, 3.01]**							
Total events	44		73										
Heterogeneity: Tau² = 0.00; Chi² = 5.75, df = 7 (P = 0.57); I² = 0%													
Test for overall effect: Z = 2.46 (P = 0.01)													
**1.23.2 EEG Asymmetries**													
Arntsen 2017	15	19	18	21	6.0%	0.63 [0.12, 3.24]	−	−	+	+	?	?	?
Asadi-Pooya 2022	65	82	42	53	10.4%	1.00 [0.43, 2.35]	−	−	?	?	+	−	−
AYKUTLU 2004	0	7	13	81	2.7%	0.34 [0.02, 6.28]	−	−	?	?	+	+	?
FERNANDO-DONGAS 2000	4	10	2	23	5.0%	7.00 [1.02, 47.97]	−	−	?	?	+	?	?
Gürer 2019	32	83	10	132	10.8%	7.65 [3.50, 16.73]	−	−	?	?	+	+	+
Lim 2023	20	25	10	24	7.8%	5.60 [1.57, 19.99]	−	−	−	?	+	?	−
Manue 2015	3	30	3	160	6.0%	5.81 [1.11, 30.33]	?	−	−	−	+		+
Senf 2013	1	27	5	39	4.1%	0.26 [0.03, 2.38]	−	−	−	−	+	+	?
**Subtotal (95% CI)**		**283**		**533**	**52.9%**	**2.16 [0.84, 5.50]**							
Total events	140		103										
Heterogeneity: Tau² = 1.16; Chi² = 24.34, df = 7 (P = 0.0010); I² = 71%													
Test for overall effect: Z = 1.61 (P = 0.11)													
**Total (95% CI)**		**494**		**1131**	**100.0%**	**1.98 [1.16, 3.38]**							
Total events	184		176										
Heterogeneity: Tau² = 0.53; Chi² = 31.45, df = 15 (P = 0.008); I² = 52%													
Test for overall effect: Z = 2.51 (P = 0.01)													
Test for subgroup differences: Chi² = 0.08, df = 1 (P = 0.77), I² = 0%													

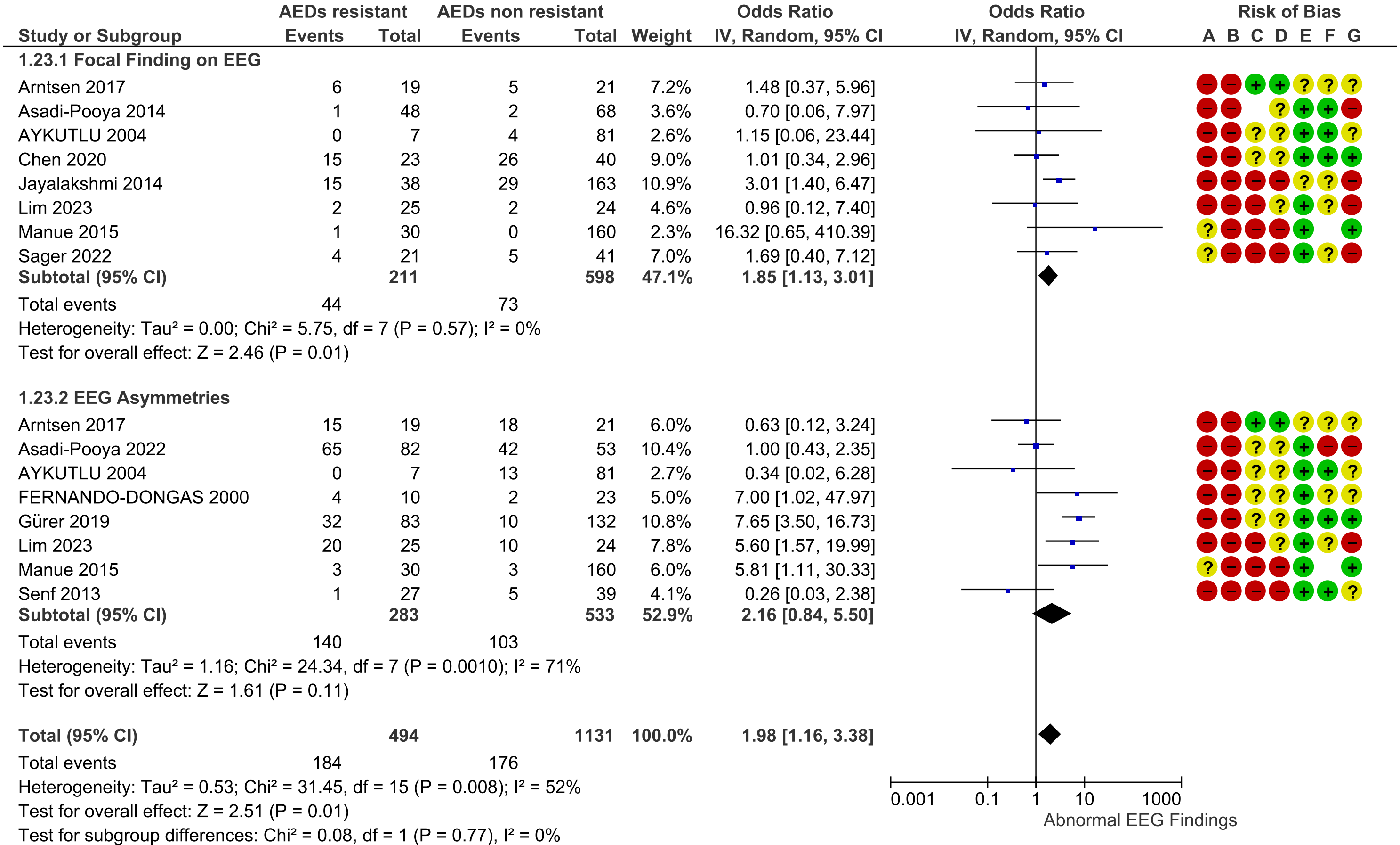

Risk of bias legend

(**A**) Random sequence generation (selection bias)
(**B**) Allocation concealment (selection bias)
(**C**) Blinding of participants and personnel (performance bias)
(**D**) Blinding of outcome assessment (detection bias)
(**E**) Incomplete outcome data (attrition bias)
(**F**) Selective reporting (reporting bias)
(**G**) Other bias

Study or Subgroup	Resistant Seizures Events	Resistant Seizures Total	Controlled Seizures Events	Controlled Seizures Total	Weight	Odds Ratio IV, Random, 95% CI
**1.12.1 CAE evolving into JME**						
Cação 2018	12	121	2	119	16.8%	6.44 [1.41, 29.43]
Gürer 2019	6	83	3	132	17.3%	3.35 [0.81, 13.78]
**Subtotal (95% CI)**		**204**		**251**	**34.2%**	**4.54 [1.61, 12.78]**
Total events	18		5			
Heterogeneity: Tau² = 0.00; Chi² = 0.38, df = 1 (P = 0.54); I² = 0%						
Test for overall effect: Z = 2.86 (P = 0.004)						
**1.12.2 JME with adolescent Abs**						
Cação 2018	6	121	3	119	17.4%	2.02 [0.49, 8.26]
**Subtotal (95% CI)**		**121**		**119**	**17.4%**	**2.02 [0.49, 8.26]**
Total events	6		3			
Heterogeneity: Not applicable						
Test for overall effect: Z = 0.98 (P = 0.33)						
**1.12.3 JME with astatic sz**						
Cação 2018	5	121	0	119	10.5%	11.28 [0.62, 206.36]
**Subtotal (95% CI)**		**121**		**119**	**10.5%**	**11.28 [0.62, 206.36]**
Total events	5		0			
Heterogeneity: Not applicable						
Test for overall effect: Z = 1.63 (P = 0.10)						
**1.12.4 Classic JME**						
Cação 2018	98	121	114	119	19.3%	0.19 [0.07, 0.51]
Hernández-Vanegas 2016	52	57	34	46	18.7%	3.67 [1.19, 11.35]
**Subtotal (95% CI)**		**178**		**165**	**38.0%**	**0.82 [0.04, 15.15]**
Total events	150		148			
Heterogeneity: Tau² = 4.14; Chi² = 14.92, df = 1 (P = 0.0001); I² = 93%						
Test for overall effect: Z = 0.13 (P = 0.89)						
**Total (95% CI)**		**624**		**654**	**100.0%**	**2.27 [0.61, 8.38]**
Total events	179		156			
Heterogeneity: Tau² = 2.04; Chi² = 25.73, df = 5 (P = 0.0001); I² = 81%						
Test for overall effect: Z = 1.23 (P = 0.22)						
Test for subgroup differences: Chi² = 2.39, df = 3 (P = 0.49), I² = 0%						

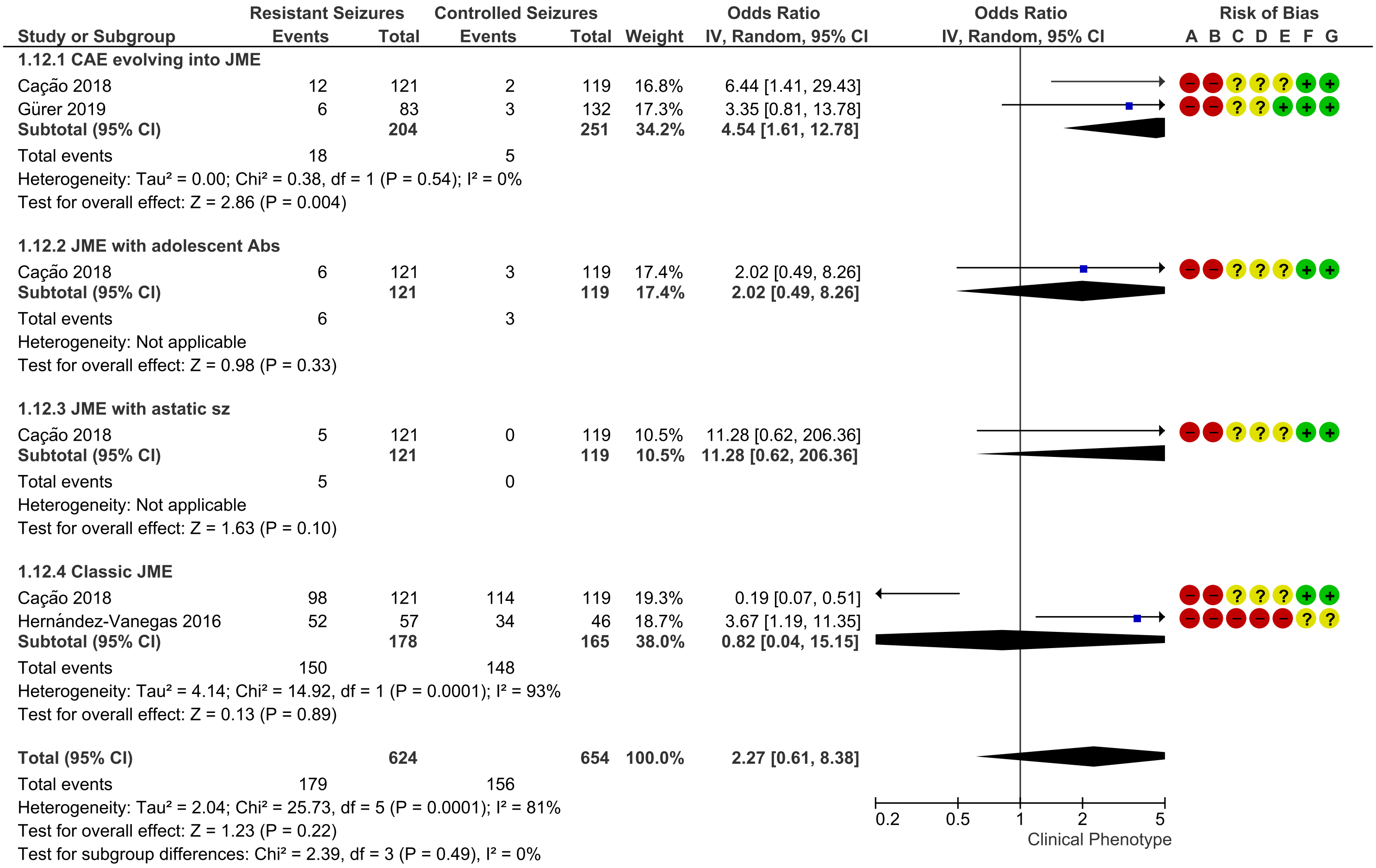

Risk of bias legend
(**A**) Random sequence generation (selection bias)
(**B**) Allocation concealment (selection bias)
(**C**) Blinding of participants and personnel (performance bias)
(**D**) Blinding of outcome assessment (detection bias)
(**E**) Incomplete outcome data (attrition bias)
(**F**) Selective reporting (reporting bias)
(**G**) Other bias

Study or Subgroup	Resistant Events	Resistant Total	Control Events	Control Total	Weight	Odds Ratio IV, Fixed, 95% CI	Risk of Bias A	B	C	D	E	F	G
Arntsen 2017	15	19	14	21	12.7%	1.88 [0.45, 7.82]	−	−	+	+	?	?	?
Asadi-Pooya 2014	2	48	4	68	8.6%	0.70 [0.12, 3.96]	−	−		?	+	+	−
Cação 2018	15	121	24	119	52.7%	0.56 [0.28, 1.13]	−	−	?	?	?	+	+
Manue 2015	2	30	3	160	7.7%	3.74 [0.60, 23.39]	?	−	−	−	+		+
MARTINOVIC´ 2001	17	22	19	36	18.3%	3.04 [0.92, 10.03]	?	−	?	?	?	?	+
**Total (95% CI)**		**240**		**404**	**100.0%**	**1.05 [0.63, 1.75]**							
Total events	51		64										

Heterogeneity: Chi² = 8.82, df = 4 (P = 0.07); I² = 55%
Test for overall effect: Z = 0.19 (P = 0.85)

Odds Ratio
IV, Fixed, 95% CI

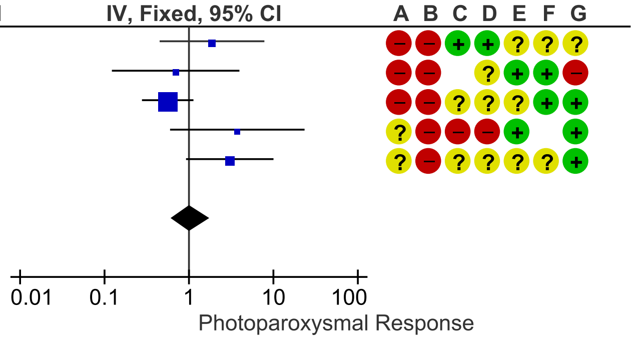

Risk of bias legend

(**A**) Random sequence generation (selection bias)
(**B**) Allocation concealment (selection bias)
(**C**) Blinding of participants and personnel (performance bias)
(**D**) Blinding of outcome assessment (detection bias)
(**E**) Incomplete outcome data (attrition bias)
(**F**) Selective reporting (reporting bias)
(**G**) Other bias

	Resistant Seizures		Controlled Seizures			Odds Ratio	Odds Ratio	Risk of Bias
**Study or Subgroup**	**Events**	**Total**	**Events**	**Total**	**Weight**	**IV, Fixed, 95% CI**	**IV, Fixed, 95% CI**	**A B C D E F G**
Gürer 2019	17	83	15	132	93.2%	2.01 [0.94, 4.28]		
Manue 2015	1	30	1	160	6.8%	5.48 [0.33, 90.15]		
**Total (95% CI)**		**113**		**292**	**100.0%**	**2.15 [1.04, 4.47]**		
Total events	18		16					

Heterogeneity: Chi² = 0.46, df = 1 (P = 0.50); I² = 0%
Test for overall effect: Z = 2.05 (P = 0.04)

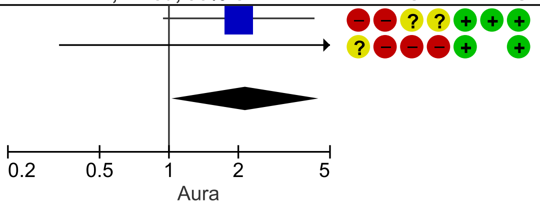

Risk of bias legend
(**A**) Random sequence generation (selection bias)
(**B**) Allocation concealment (selection bias)
(**C**) Blinding of participants and personnel (performance bias)
(**D**) Blinding of outcome assessment (detection bias)
(**E**) Incomplete outcome data (attrition bias)
(**F**) Selective reporting (reporting bias)
(**G**) Other bias

Study or Subgroup	Resistant Seizures Events	Resistant Seizures Total	Controlled Seizures Events	Controlled Seizures Total	Weight	Odds Ratio IV, Random, 95% CI	Risk of Bias A	B	C	D	E	F	G
Asadi-Pooya 2014	18	48	24	68	30.0%	1.10 [0.51, 2.37]	−	−		?	+	+	−
Asadi-Pooya 2022	35	82	29	53	32.2%	0.62 [0.31, 1.24]	−	−	?	?	+	−	−
Gürer 2019	20	83	26	132	33.2%	1.29 [0.67, 2.51]	−	−	?	?	+	+	+
Manue 2015	2	30	0	160	4.5%	28.16 [1.32, 601.98]	?	−	−	−	+		+
**Total (95% CI)**		**243**		**413**	**100.0%**	**1.12 [0.56, 2.22]**							
Total events	75		79										

Heterogeneity: Tau² = 0.25; Chi² = 7.06, df = 3 (P = 0.07); I² = 58%

Test for overall effect: Z = 0.32 (P = 0.75)

Odds Ratio IV, Random, 95% CI

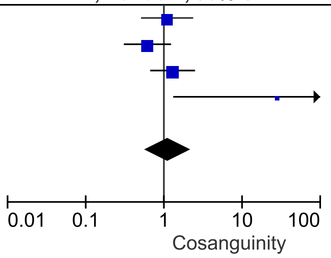

Risk of bias legend

(**A**) Random sequence generation (selection bias)
(**B**) Allocation concealment (selection bias)
(**C**) Blinding of participants and personnel (performance bias)
(**D**) Blinding of outcome assessment (detection bias)
(**E**) Incomplete outcome data (attrition bias)
(**F**) Selective reporting (reporting bias)
(**G**) Other bias

Study or Subgroup	Resistant Seizures Events	Resistant Seizures Total	Controlled Seizures Events	Controlled Seizures Total	Weight	Odds Ratio IV, Random, 95% CI	Risk of Bias A	B	C	D	E	F	G
Asadi-Pooya 2014	21	48	20	68	27.3%	1.87 [0.86, 4.04]	−	−		?	+	+	−
AYKUTLU 2004	4	7	0	81	11.1%	209.57 [9.33, 4705.74]	−	−	?	?	+	+	?
Cação 2018	105	121	108	119	27.0%	0.67 [0.30, 1.51]	−	−	?	?	?	+	+
Gürer 2019	14	83	4	132	24.5%	6.49 [2.06, 20.49]	−	−	?	?	+	+	+
Hirano 2008	0	1	4	33	10.1%	2.19 [0.08, 62.33]	−	−	−	?	?	+	−
**Total (95% CI)**		**260**		**433**	**100.0%**	**3.29 [0.89, 12.10]**							
Total events	144		136										

Heterogeneity: Tau² = 1.46; Chi² = 19.49, df = 4 (P = 0.0006); I² = 79%
Test for overall effect: Z = 1.79 (P = 0.07)

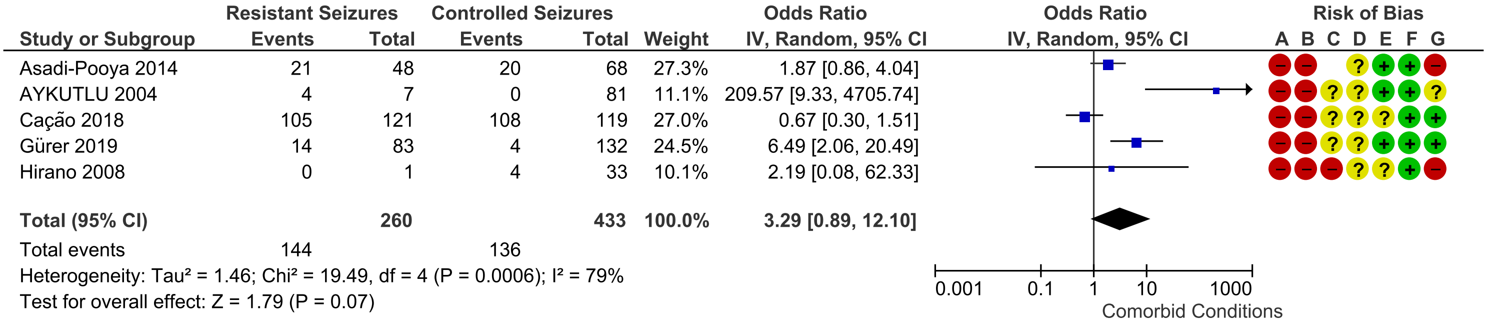

Risk of bias legend

(**A**) Random sequence generation (selection bias)
(**B**) Allocation concealment (selection bias)
(**C**) Blinding of participants and personnel (performance bias)
(**D**) Blinding of outcome assessment (detection bias)
(**E**) Incomplete outcome data (attrition bias)
(**F**) Selective reporting (reporting bias)
(**G**) Other bias

Study or Subgroup	Resistant Seizures Events	Resistant Seizures Total	Controlled Seizures Events	Controlled Seizures Total	Weight	Odds Ratio IV, Random, 95% CI	Risk of Bias A	B	C	D	E	F	G
Asadi-Pooya 2014	5	48	0	68	54.9%	17.32 [0.93, 321.13]	−	−		?	+	+	−
Lim 2023	0	25	1	24		Not estimable	−	−	−	?	+	?	−
Manue 2015	1	30	0	160	45.1%	16.32 [0.65, 410.39]	?	−	−	−	+		+
**Total (95% CI)**		**78**		**228**	**100.0%**	**16.86 [1.94, 146.88]**							
Total events	6		0										

Heterogeneity: Tau² = 0.00; Chi² = 0.00, df = 1 (P = 0.98); I² = 0%
Test for overall effect: Z = 2.56 (P = 0.01)

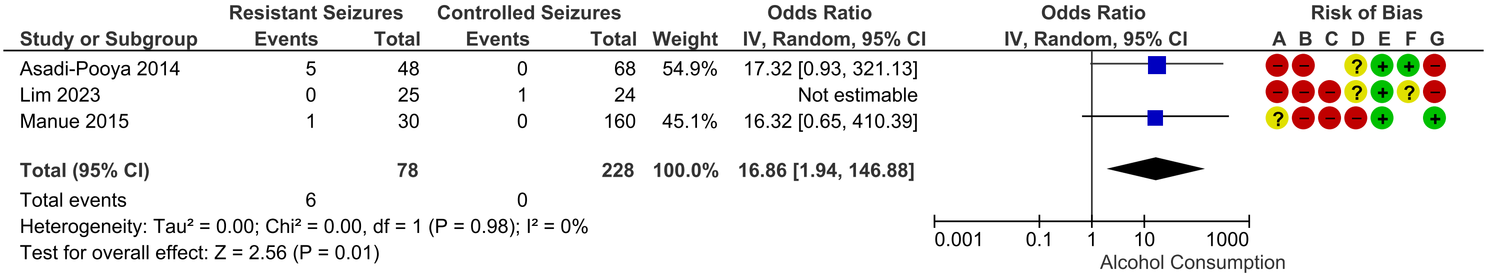

Risk of bias legend
(**A**) Random sequence generation (selection bias)
(**B**) Allocation concealment (selection bias)
(**C**) Blinding of participants and personnel (performance bias)
(**D**) Blinding of outcome assessment (detection bias)
(**E**) Incomplete outcome data (attrition bias)
(**F**) Selective reporting (reporting bias)
(**G**) Other bias

Study or Subgroup	Resistant Mean	Resistant SD	Resistant Total	Control Mean	Control SD	Control Total	Weight	Mean Difference IV, Fixed, 95% CI	A	B	C	D	E	F	G
Arntsen 2017	31.9	7.1	19	31.6	10.5	21	1.2%	0.30 [-5.21, 5.81]	−	−	+	+	?	?	?
Asadi-Pooya 2014	33.2	10.3	48	37.2	8.4	68	3.0%	-4.00 [-7.53, -0.47]	−	−		?	+	+	−
Asadi-Pooya 2022	11	2	82	11	2	53	77.4%	0.00 [-0.69, 0.69]	−	−	?	?	+	−	−
Gürer 2019	10.62	5.72	83	9.59	5.26	132	15.9%	1.03 [-0.49, 2.55]	−	−	?	?	+	+	+
Jayalakshmi 2014	86.4	26.6	38	85.4	25.6	163	0.4%	1.00 [-8.33, 10.33]	−	−	−	−	?	?	−
Lim 2023	11.8	10.6	25	11	7.9	24	1.4%	0.80 [-4.42, 6.02]	−	−	−	?	+	?	−
Senf 2013	44.5	15	27	44.7	13.1	39	0.8%	-0.20 [-7.19, 6.79]	−	−	−	−	+	+	?
**Total (95% CI)**			**322**			**500**	**100.0%**	**0.06 [-0.54, 0.67]**							

Heterogeneity: Chi² = 6.79, df = 6 (P = 0.34); I² = 12%
Test for overall effect: Z = 0.20 (P = 0.84)

Mean Difference IV, Fixed, 95% CI

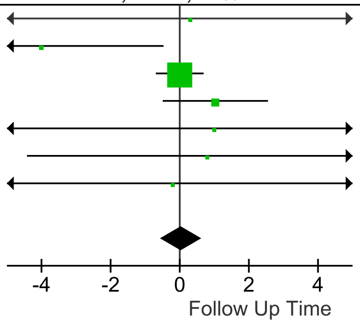

Risk of bias legend
(**A**) Random sequence generation (selection bias)
(**B**) Allocation concealment (selection bias)
(**C**) Blinding of participants and personnel (performance bias)
(**D**) Blinding of outcome assessment (detection bias)
(**E**) Incomplete outcome data (attrition bias)
(**F**) Selective reporting (reporting bias)
(**G**) Other bias

Study or Subgroup	Resistant Seizures Events	Resistant Seizures Total	Controlled Seizures Events	Controlled Seizures Total	Weight	Odds Ratio IV, Random, 95% CI	A	B	C	D	E	F	G
AYKUTLU 2004	0	7	36	81	7.8%	0.08 [0.00, 1.50]	−	−	?	?	+	+	?
Gürer 2019	11	83	20	132	26.7%	0.86 [0.39, 1.89]	−	−	?	?	+	+	+
Lim 2023	2	25	6	24	15.5%	0.26 [0.05, 1.45]	−	−	−	?	+	?	−
Manue 2015	3	30	0	160	7.4%	40.85 [2.05, 812.96]	?	−	−	−	+		+
Sager 2022	6	21	16	41	22.1%	0.63 [0.20, 1.95]	?	−	−	−	+	?	−
Senf 2013	4	27	12	39	20.5%	0.39 [0.11, 1.38]	−	−	−	−	+	+	?
**Total (95% CI)**		**193**		**477**	**100.0%**	**0.63 [0.25, 1.58]**							
Total events	26		90										

Odds Ratio IV, Random, 95% CI

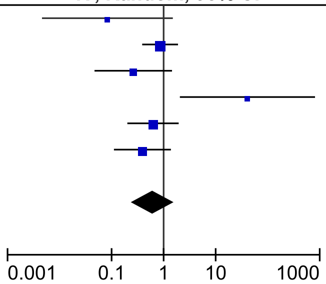

Heterogeneity: Tau² = 0.66; Chi² = 11.48, df = 5 (P = 0.04); I² = 56%

Test for overall effect: Z = 0.99 (P = 0.32)

Risk of bias legend

(**A**) Random sequence generation (selection bias)

(**B**) Allocation concealment (selection bias)

(**C**) Blinding of participants and personnel (performance bias)

(**D**) Blinding of outcome assessment (detection bias)

(**E**) Incomplete outcome data (attrition bias)

(**F**) Selective reporting (reporting bias)

(**G**) Other bias

Study or Subgroup	Resistant Seizures Events	Resistant Seizures Total	Controlled Seizures Events	Controlled Seizures Total	Weight	Odds Ratio IV, Random, 95% CI	Risk of Bias A	B	C	D	E	F	G
**1.8.1 No**													
Lim 2023	8	25	18	24	33.9%	0.16 [0.04, 0.55]	−	−	−	?	+	?	−
Senf 2013	18	27	22	39	36.4%	1.55 [0.56, 4.29]	−	−	−	−	+	+	?
**Subtotal (95% CI)**		**52**		**63**	**70.3%**	**0.51 [0.05, 4.77]**							
Total events	26		40										
Heterogeneity: Tau² = 2.28; Chi² = 7.73, df = 1 (P = 0.005); I² = 87%													
Test for overall effect: Z = 0.59 (P = 0.55)													
**1.8.2 Yes**													
Sánchez-Zapata 2019	49	51	87	94	29.7%	1.97 [0.39, 9.86]	?	?	+	?	?	?	−
**Subtotal (95% CI)**		**51**		**94**	**29.7%**	**1.97 [0.39, 9.86]**							
Total events	49		87										
Heterogeneity: Not applicable													
Test for overall effect: Z = 0.83 (P = 0.41)													
**Total (95% CI)**		**103**		**157**	**100.0%**	**0.77 [0.16, 3.76]**							
Total events	75		127										
Heterogeneity: Tau² = 1.54; Chi² = 9.33, df = 2 (P = 0.009); I² = 79%													
Test for overall effect: Z = 0.33 (P = 0.74)													
Test for subgroup differences: Chi² = 0.93, df = 1 (P = 0.33), I² = 0%													

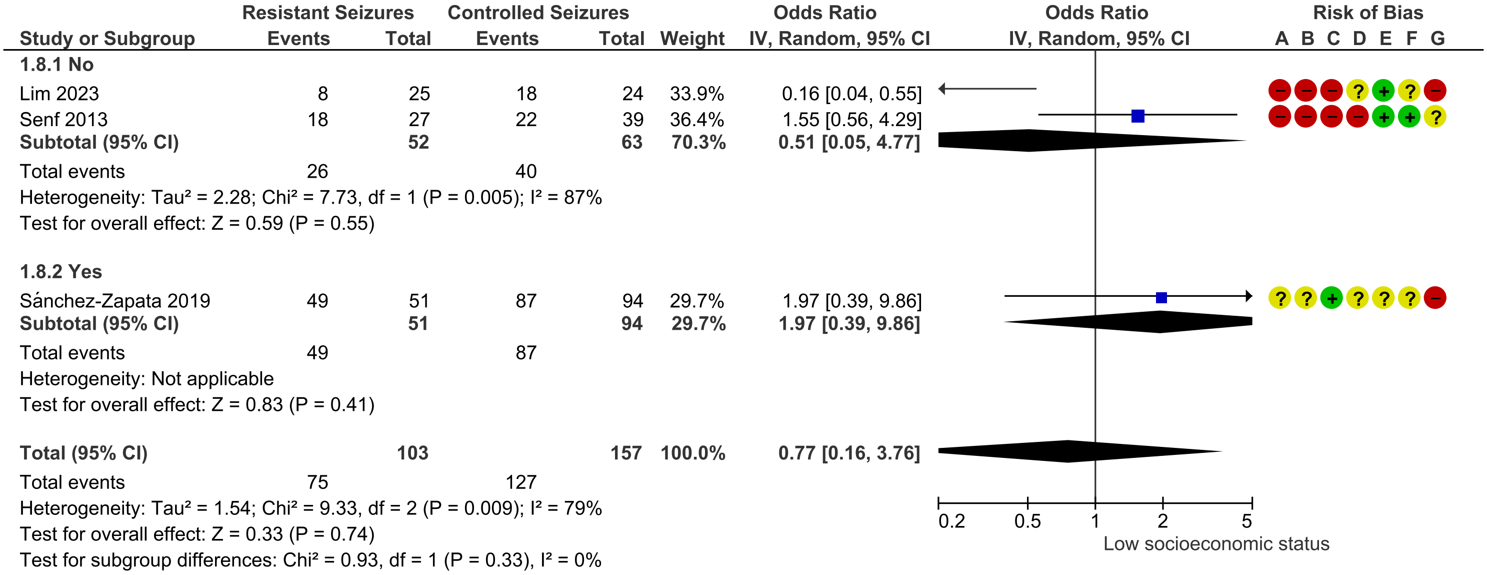

Risk of bias legend
(**A**) Random sequence generation (selection bias)
(**B**) Allocation concealment (selection bias)
(**C**) Blinding of participants and personnel (performance bias)
(**D**) Blinding of outcome assessment (detection bias)
(**E**) Incomplete outcome data (attrition bias)
(**F**) Selective reporting (reporting bias)
(**G**) Other bias

Study or Subgroup	Resistant Seizures Events	Resistant Seizures Total	Controlled Seizures Events	Controlled Seizures Total	Weight	Odds Ratio IV, Fixed, 95% CI	A	B	C	D	E	F	G
**1.3.1 Male**													
Asadi-Pooya 2014	16	48	22	68	3.1%	1.05 [0.48, 2.30]	−	−		?	+	+	−
Asadi-Pooya 2022	29	82	14	53	3.4%	1.52 [0.71, 3.26]	−	−	?	?	+	−	−
AYKUTLU 2004	5	7	29	81	0.7%	4.48 [0.82, 24.58]	−	−	?	?	+	+	?
Cação 2018	41	121	53	119	7.1%	0.64 [0.38, 1.08]	−	−	?	?	?	+	+
FERNANDO-DONGAS 2000	5	10	10	23	0.9%	1.30 [0.29, 5.76]	−	−	?	?	+	?	?
Gürer 2019	41	83	53	132	6.4%	1.46 [0.84, 2.53]	−	−	?	?	+	+	+
Hernández-Vanegas 2016	27	57	20	46	3.2%	1.17 [0.54, 2.56]	−	−	−	−	−	?	?
Hirano 2008	0	1	13	33	0.2%	0.51 [0.02, 13.36]	−	−	−	?	?	+	−
Jayalakshmi 2014	22	38	89	163	3.8%	1.14 [0.56, 2.33]	−	−	−	−	?	?	−
MARTINOVIC´ 2001	10	22	18	36	1.7%	0.83 [0.29, 2.41]	?	−	?	?	?	?	+
Sager 2022	8	21	13	41	1.6%	1.33 [0.44, 3.98]	?	−	−	−	+	?	−
Sánchez-Zapata 2019	11	27	22	39	2.0%	0.53 [0.20, 1.44]	?	?	+	?	?	?	−
Senf 2013	11	27	22	39	2.0%	0.53 [0.20, 1.44]	−	−	−	−	+	+	?
Shakeshaft 2022	52	165	127	342	12.5%	0.78 [0.52, 1.16]	−	−	−	?	?	?	?
Viswanathan 2021	11	22	15	34	1.7%	1.27 [0.43, 3.71]	−	−	−	?	−	?	−
Yam 2022	3	8	4	11	0.5%	1.05 [0.16, 6.92]	−	−	?	?	?	?	?
**Subtotal (95% CI)**		**739**		**1260**	**50.8%**	**0.96 [0.79, 1.17]**							
Total events	292		524										
Heterogeneity: Chi² = 14.37, df = 15 (P = 0.50); I² = 0%													
Test for overall effect: Z = 0.43 (P = 0.67)													
**1.3.2 Female**													
Arntsen 2017	14	19	11	21	1.1%	2.55 [0.67, 9.65]	−	−	+	+	?	?	?
Asadi-Pooya 2014	32	48	46	68	3.1%	0.96 [0.44, 2.10]	−	−		?	+	+	−
Asadi-Pooya 2022	53	82	39	53	3.4%	0.66 [0.31, 1.40]	−	−	?	?	+	−	−
AYKUTLU 2004	2	7	52	81	0.7%	0.22 [0.04, 1.22]	−	−	?	?	+	+	?
Cação 2018	80	121	66	119	7.1%	1.57 [0.93, 2.64]	−	−	?	?	?	+	+
Chen 2020	11	23	19	40	1.8%	1.01 [0.36, 2.83]	−	−	?	?	+	+	+
FERNANDO-DONGAS 2000	5	10	13	23	0.9%	0.77 [0.17, 3.41]	−	−	?	?	+	?	?
Gürer 2019	42	83	79	132	6.4%	0.69 [0.40, 1.20]	−	−	?	?	+	+	+
Hernández-Vanegas 2016	30	57	26	46	3.2%	0.85 [0.39, 1.87]	−	−	−	−	−	?	?
Hirano 2008	1	1	20	33	0.2%	1.98 [0.07, 52.16]	−	−	−	?	?	+	−
Lim 2023	11	25	14	24	1.5%	0.56 [0.18, 1.74]	−	−	−	?	+	?	−
Martin 2019	8	19	35	61	1.8%	0.54 [0.19, 1.53]	?	−	?	?	−	+	+
MARTINOVIC´ 2001	12	22	18	36	1.7%	1.20 [0.41, 3.48]	?	−	?	?	?	?	+
Sager 2022	13	21	28	41	1.6%	0.75 [0.25, 2.26]	?	−	−	−	+	?	−
Shakeshaft 2022	113	165	215	342	12.5%	1.28 [0.86, 1.91]	−	−	−	?	?	?	?
Viswanathan 2021	11	22	19	34	1.7%	0.79 [0.27, 2.31]	−	−	−	?	−	?	−
Yam 2022	5	8	7	11	0.5%	0.95 [0.14, 6.28]	−	−	?	?	?	?	?
**Subtotal (95% CI)**		**733**		**1165**	**49.2%**	**0.99 [0.81, 1.20]**							
Total events	443		707										
Heterogeneity: Chi² = 15.52, df = 16 (P = 0.49); I² = 0%													
Test for overall effect: Z = 0.15 (P = 0.88)													
**Total (95% CI)**		**1472**		**2425**	**100.0%**	**0.97 [0.84, 1.12]**							
Total events	735		1231										
Heterogeneity: Chi² = 29.94, df = 32 (P = 0.57); I² = 0%													
Test for overall effect: Z = 0.41 (P = 0.68)													
Test for subgroup differences: Chi² = 0.04, df = 1 (P = 0.84), I² = 0%													

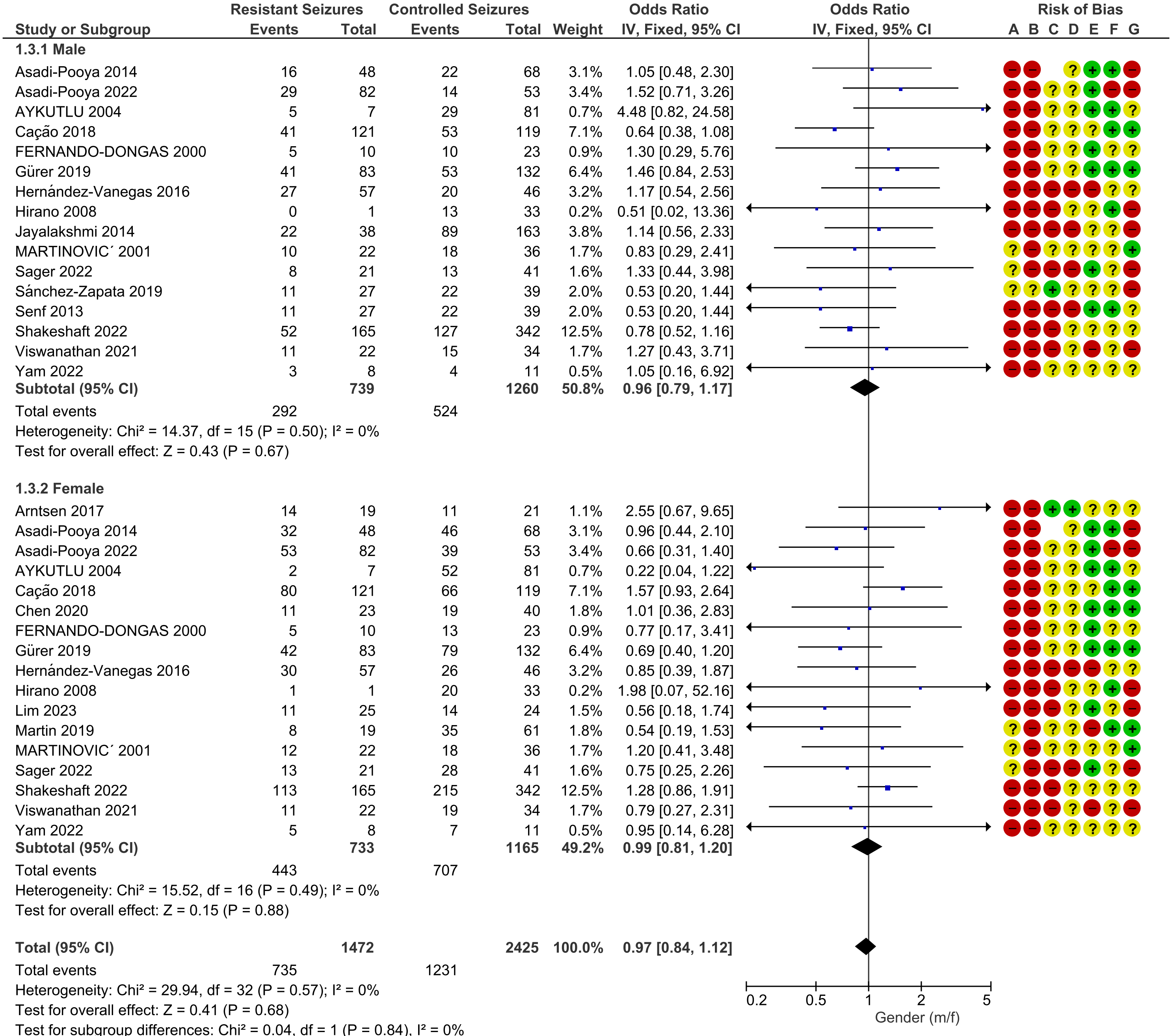

Risk of bias legend

(**A**) Random sequence generation (selection bias)
(**B**) Allocation concealment (selection bias)
(**C**) Blinding of participants and personnel (performance bias)
(**D**) Blinding of outcome assessment (detection bias)
(**E**) Incomplete outcome data (attrition bias)
(**F**) Selective reporting (reporting bias)
(**G**) Other bias

Study or Subgroup	Resistant Seizures Events	Resistant Seizures Total	Controlled Seizures Events	Controlled Seizures Total	Weight	Odds Ratio IV, Fixed, 95% CI	A	B	C	D	E	F	G
Asadi-Pooya 2014	1	48	3	68	4.3%	0.46 [0.05, 4.57]	−	−		?	+	+	−
AYKUTLU 2004	0	7	14	81	2.7%	0.31 [0.02, 5.74]	−	−	?	?	+	+	?
Chen 2020	6	23	3	40	10.1%	4.35 [0.97, 19.51]	−	−	?	?	+	+	+
Gürer 2019	17	83	18	132	43.0%	1.63 [0.79, 3.38]	−	−	?	?	+	+	+
Hernández-Vanegas 2016	2	57	2	46	5.7%	0.80 [0.11, 5.91]	−	−	−	−	−	?	?
Jayalakshmi 2014	3	38	21	163	14.3%	0.58 [0.16, 2.05]	−	−	−	−	?	?	−
Lim 2023	5	25	1	24	4.6%	5.75 [0.62, 53.43]	−	−	−	?	+	?	−
Manue 2015	0	30	1	160	2.2%	1.74 [0.07, 43.80]	?	−	−	−	+		+
Martin 2019	2	19	0	61	2.4%	17.57 [0.81, 383.26]	?	−	?	?	−	+	+
Sager 2022	9	21	3	41	10.7%	9.50 [2.21, 40.86]	?	−	−	−	+	?	−
**Total (95% CI)**		**351**		**816**	**100.0%**	**1.83 [1.14, 2.96]**							
Total events	45		66										

Heterogeneity: Chi² = 15.99, df = 9 (P = 0.07); I² = 44%
Test for overall effect: Z = 2.49 (P = 0.01)

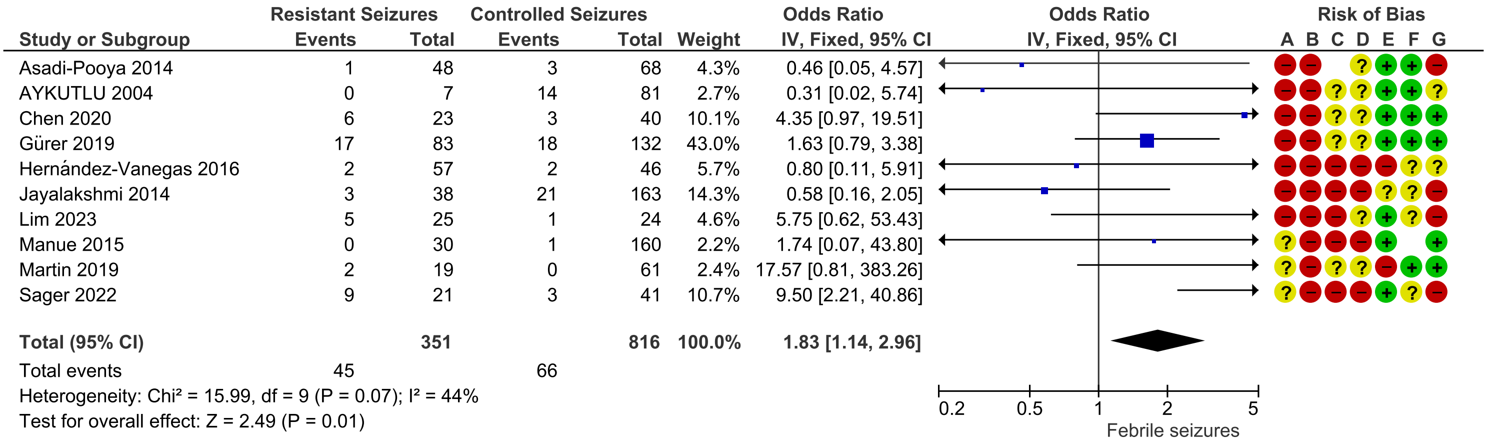

Risk of bias legend

(**A**) Random sequence generation (selection bias)
(**B**) Allocation concealment (selection bias)
(**C**) Blinding of participants and personnel (performance bias)
(**D**) Blinding of outcome assessment (detection bias)
(**E**) Incomplete outcome data (attrition bias)
(**F**) Selective reporting (reporting bias)
(**G**) Other bias

Study or Subgroup	Resistant Seizures Events	Resistant Seizures Total	Controlled Seizures Events	Controlled Seizures Total	Weight	Odds Ratio IV, Random, 95% CI	A	B	C	D	E	F	G
Asadi-Pooya 2014	0	48	5	68	23.6%	0.12 [0.01, 2.20]	−	−		?	+	+	−
Asadi-Pooya 2022	4	82	0	53	23.5%	6.13 [0.32, 116.30]	−	−	?	?	+	−	−
AYKUTLU 2004	4	7	0	81	22.7%	209.57 [9.33, 4705.74]	−	−	?	?	+	+	?
Gürer 2019	8	83	2	132	30.2%	6.93 [1.43, 33.50]	−	−	?	?	+	+	+
**Total (95% CI)**		**220**		**334**	**100.0%**	**5.59 [0.43, 71.98]**							
Total events	16		7										

Heterogeneity: Tau² = 4.98; Chi² = 11.97, df = 3 (P = 0.008); I² = 75%
Test for overall effect: Z = 1.32 (P = 0.19)

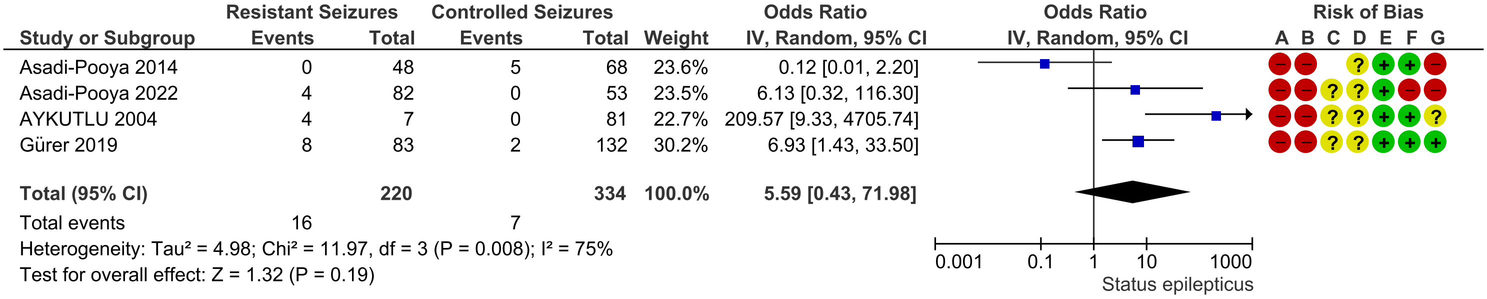

Risk of bias legend
(**A**) Random sequence generation (selection bias)
(**B**) Allocation concealment (selection bias)
(**C**) Blinding of participants and personnel (performance bias)
(**D**) Blinding of outcome assessment (detection bias)
(**E**) Incomplete outcome data (attrition bias)
(**F**) Selective reporting (reporting bias)
(**G**) Other bias

Study or Subgroup	Resistant Seizures Events	Resistant Seizures Total	Controlled Seizures Events	Controlled Seizures Total	Weight	Odds Ratio IV, Fixed, 95% CI
**1.2.1 Depressive Disorders**						
Gelisse 2001	2	24	1	116	1.5%	10.45 [0.91, 120.35]
Martin 2019	9	19	15	61	7.8%	2.76 [0.94, 8.07]
**Subtotal (95% CI)**		**43**		**177**	**9.3%**	**3.42 [1.28, 9.14]**
Total events	11		16			
Heterogeneity: Chi² = 0.96, df = 1 (P = 0.33); I² = 0%						
Test for overall effect: Z = 2.46 (P = 0.01)						
**1.2.2 Anxiety**						
Gelisse 2001	3	24	3	116	3.2%	5.38 [1.02, 28.49]
Guaranha 2011	32	40	16	25	7.0%	2.25 [0.73, 6.94]
**Subtotal (95% CI)**		**64**		**141**	**10.3%**	**2.96 [1.16, 7.52]**
Total events	35		19			
Heterogeneity: Chi² = 0.72, df = 1 (P = 0.40); I² = 0%						
Test for overall effect: Z = 2.28 (P = 0.02)						
**1.2.3 Psychotic Disorders**						
Gelisse 2001	0	24	2	116	0.9%	0.93 [0.04, 20.09]
Martin 2019	1	19	0	61	0.8%	9.97 [0.39, 255.29]
**Subtotal (95% CI)**		**43**		**177**	**1.8%**	**2.86 [0.31, 26.55]**
Total events	1		2			
Heterogeneity: Chi² = 1.08, df = 1 (P = 0.30); I² = 7%						
Test for overall effect: Z = 0.92 (P = 0.36)						
**1.2.4 Mental Retardation**						
Gelisse 2001	0	24	1	116	0.9%	1.57 [0.06, 39.73]
**Subtotal (95% CI)**		**24**		**116**	**0.9%**	**1.57 [0.06, 39.73]**
Total events	0		1			
Heterogeneity: Not applicable						
Test for overall effect: Z = 0.27 (P = 0.78)						
**1.2.5 Psychiatric History (not defiened)**						
AYKUTLU 2004	3	7	4	81	2.7%	14.44 [2.38, 87.61]
Cação 2018	17	121	9	119	12.3%	2.00 [0.85, 4.68]
Gelisse 2001	14	24	22	116	10.2%	5.98 [2.35, 15.24]
Gürer 2019	20	83	14	132	15.9%	2.68 [1.27, 5.66]
Hernández-Vanegas 2016	8	57	2	46	3.5%	3.59 [0.72, 17.83]
Hirano 2008	0	1	2	33	0.8%	4.20 [0.13, 132.30]
Jayalakshmi 2014	25	38	42	163	15.6%	5.54 [2.60, 11.81]
Manue 2015	2	30	0	160	1.0%	28.16 [1.32, 601.98]
Sánchez-Zapata 2019	5	51	8	94	6.5%	1.17 [0.36, 3.78]
**Subtotal (95% CI)**		**412**		**944**	**68.5%**	**3.52 [2.45, 5.05]**
Total events	94		103			
Heterogeneity: Chi² = 12.36, df = 8 (P = 0.14); I² = 35%						
Test for overall effect: Z = 6.84 (P < 0.00001)						
**1.2.6 Personality Disorders**						
Gelisse 2001	6	24	12	116	7.4%	2.89 [0.96, 8.68]
Guaranha 2011	10	40	1	25	2.0%	8.00 [0.96, 66.95]
**Subtotal (95% CI)**		**64**		**141**	**9.4%**	**3.58 [1.35, 9.52]**
Total events	16		13			
Heterogeneity: Chi² = 0.70, df = 1 (P = 0.40); I² = 0%						
Test for overall effect: Z = 2.56 (P = 0.01)						
**Total (95% CI)**		**650**		**1696**	**100.0%**	**3.42 [2.54, 4.61]**
Total events	157		154			
Heterogeneity: Chi² = 16.19, df = 17 (P = 0.51); I² = 0%						
Test for overall effect: Z = 8.07 (P < 0.00001)						
Test for subgroup differences: Chi² = 0.38, df = 5 (P = 1.00), I² = 0%						

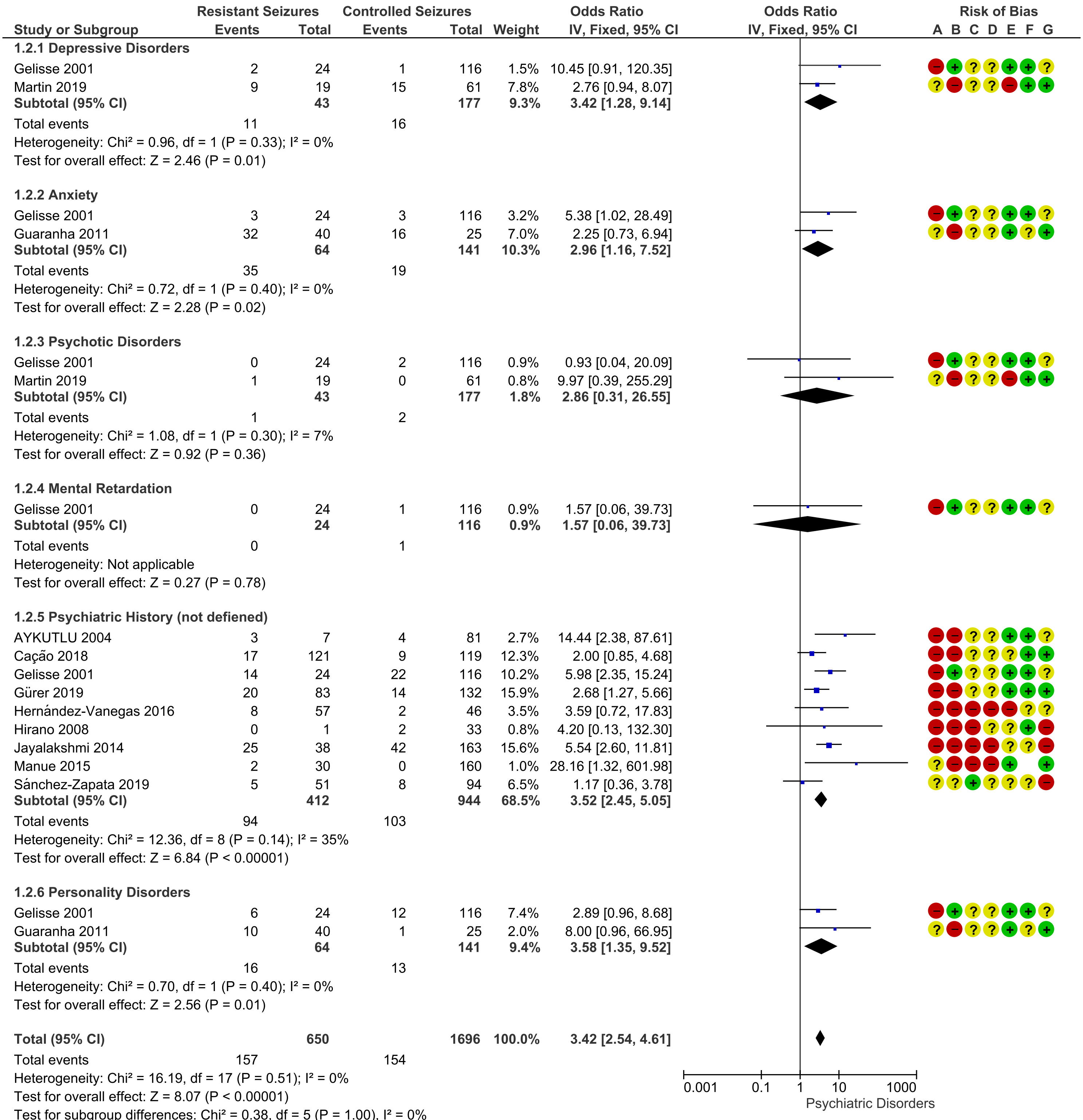

Risk of bias legend

(**A**) Random sequence generation (selection bias)
(**B**) Allocation concealment (selection bias)
(**C**) Blinding of participants and personnel (performance bias)
(**D**) Blinding of outcome assessment (detection bias)
(**E**) Incomplete outcome data (attrition bias)
(**F**) Selective reporting (reporting bias)
(**G**) Other bias

Study or Subgroup	Resistant Seizures Mean	SD	Total	Controlled Seizures Mean	SD	Total	Weight	Mean Difference IV, Fixed, 95% CI
Asadi-Pooya 2014	24.9	8.8	48	23.1	7.5	68	13.0%	1.80 [-1.26, 4.86]
Cação 2018	15	5.1	121	16	4.7	119	79.2%	-1.00 [-2.24, 0.24]
FERNANDO-DONGAS 2000	11.6	0	10	8.9	0	23		Not estimable
Lim 2023	22.6	10.3	25	19.6	6.5	24	5.3%	3.00 [-1.80, 7.80]
MARTINOVIC´ 2001	20.3	0	22	18.5	0	36		Not estimable
Senf 2013	58.5	14.6	27	59.2	13.4	39	2.5%	-0.70 [-7.63, 6.23]
**Total (95% CI)**			**253**			**309**	**100.0%**	**-0.42 [-1.52, 0.69]**

Heterogeneity: Chi² = 4.81, df = 3 (P = 0.19); I² = 38%
Test for overall effect: Z = 0.74 (P = 0.46)

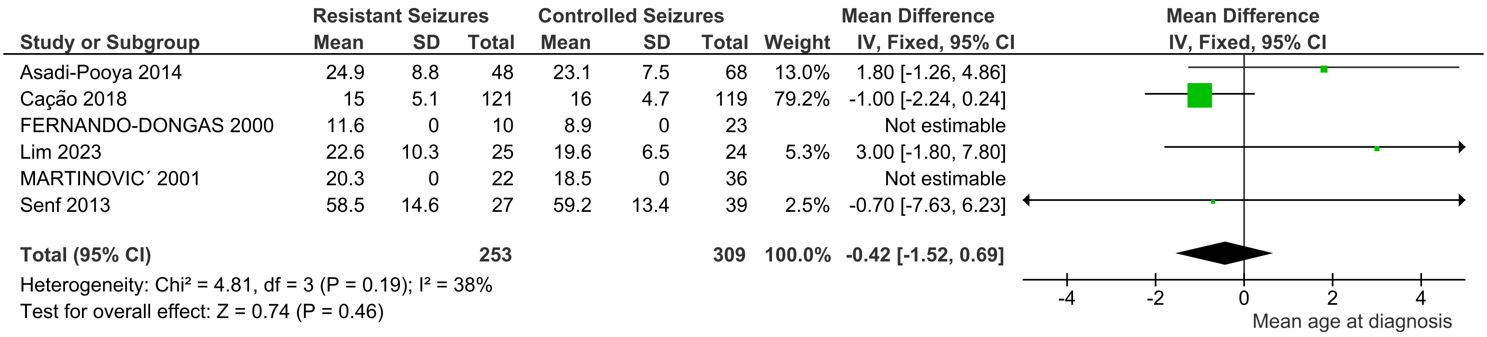

Study or Subgroup	Resistant Seizures Mean	Resistant Seizures SD	Resistant Seizures Total	Controlled Seizures Mean	Controlled Seizures SD	Controlled Seizures Total	Weight	Mean Difference IV, Fixed, 95% CI	Risk of Bias A	B	C	D	E	F	G
Arntsen 2017	14.2	2.9	19	16.2	3.9	21	3.9%	-2.00 [-4.12, 0.12]	−	−	+	+	?	?	?
Asadi-Pooya 2014	16	3.2	48	15.4	4.6	68	8.7%	0.60 [-0.82, 2.02]	−	−		?	+	+	−
Asadi-Pooya 2022	15	5	83	15	5	53	5.9%	0.00 [-1.72, 1.72]	−	−	?	?	+	−	−
AYKUTLU 2004	12.7	3.4	7	14	3.4	81	2.5%	-1.30 [-3.93, 1.33]	−	−	?	?	+	+	?
Cação 2018	14	4.7	121	15	4.1	119	14.1%	-1.00 [-2.12, 0.12]	−	−	?	?	?	+	+
FERNANDO-DONGAS 2000	14	0	10	16	0	23		Not estimable	−	−	?	?	+	?	?
Guaranha 2011	12.6	3.33	40	15.4	5.47	25	3.1%	-2.80 [-5.18, -0.42]	?	−	?	?	+	?	+
Gürer 2019	14.65	3.042	83	15.1	3.484	132	22.5%	-0.45 [-1.33, 0.43]	−	−	?	?	+	+	+
Hernández-Vanegas 2016	13.91	3.025	57	13.44	2.91	46	13.3%	0.47 [-0.68, 1.62]	−	−	−	−	−	?	?
Hirano 2008	14	0	1	13	2.1	33		Not estimable	−	−	−	?	?	+	−
Lim 2023	13.7	3.2	25	14.8	3	24	5.8%	-1.10 [-2.84, 0.64]	−	−	−	?	+	?	−
Manue 2015	13.4	2.81	30	12.7	2.76	160	14.7%	0.70 [-0.39, 1.79]	?	−	−	−	+		+
MARTINOVIC´ 2001	13.9	0	22	13.1	0	36		Not estimable	?	−	?	?	?	?	+
Sánchez-Zapata 2019	14	0	51	14	0	94		Not estimable	?	?	+	?	?	?	−
Senf 2013	14	5.6	27	14.6	3	39	3.3%	-0.60 [-2.91, 1.71]	−	−	−	−	+	+	?
Viswanathan 2021	13.4	6	22	15.3	4.4	34	2.1%	-1.90 [-4.81, 1.01]	−	−	−	?	−	?	−
**Total (95% CI)**			**646**			**988**	**100.0%**	**-0.35 [-0.77, 0.07]**							

Heterogeneity: Chi² = 17.49, df = 11 (P = 0.09); I² = 37%

Test for overall effect: Z = 1.62 (P = 0.11)

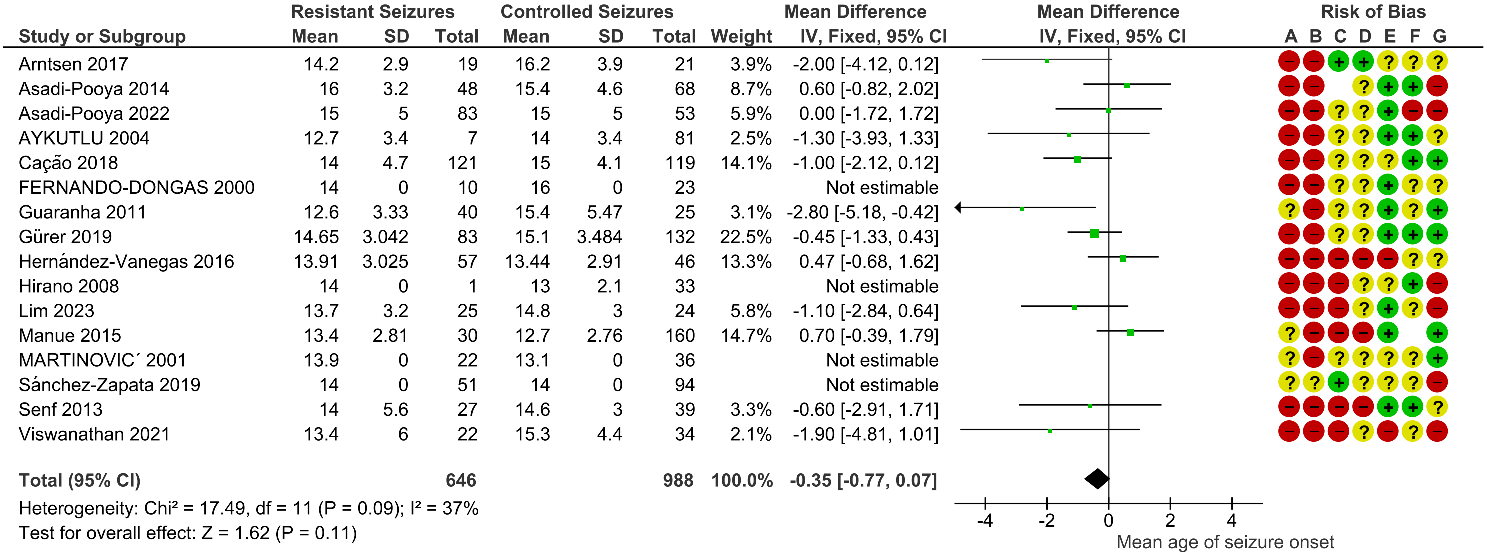

Risk of bias legend

(**A**) Random sequence generation (selection bias)

(**B**) Allocation concealment (selection bias)

(**C**) Blinding of participants and personnel (performance bias)

(**D**) Blinding of outcome assessment (detection bias)

(**E**) Incomplete outcome data (attrition bias)

(**F**) Selective reporting (reporting bias)

(**G**) Other bias

Study or Subgroup	Resistant Seizures Events	Resistant Seizures Total	Controlled Seizures Events	Controlled Seizures Total	Weight	Odds Ratio IV, Random, 95% CI
**1.6.1 Yes**						
Arntsen 2017	12	19	17	21	14.6%	0.40 [0.10, 1.69]
Asadi-Pooya 2022	42	82	29	53	21.2%	0.87 [0.43, 1.74]
Lim 2023	14	25	14	24	17.2%	0.91 [0.29, 2.82]
**Subtotal (95% CI)**		**126**		**98**	**52.9%**	**0.79 [0.45, 1.36]**
Total events	68		60			
Heterogeneity: Tau² = 0.00; Chi² = 0.98, df = 2 (P = 0.61); I² = 0%						
Test for overall effect: Z = 0.87 (P = 0.39)						
**1.6.2 No**						
FERNANDO-DONGAS 2000	2	10	0	23	5.7%	13.82 [0.60, 318.12]
Manue 2015	6	30	4	160	15.4%	9.75 [2.56, 37.09]
Martin 2019	2	19	0	61	5.9%	17.57 [0.81, 383.26]
Sánchez-Zapata 2019	11	51	23	94	20.1%	0.85 [0.38, 1.92]
**Subtotal (95% CI)**		**110**		**338**	**47.1%**	**4.99 [0.82, 30.40]**
Total events	21		27			
Heterogeneity: Tau² = 2.31; Chi² = 13.00, df = 3 (P = 0.005); I² = 77%						
Test for overall effect: Z = 1.74 (P = 0.08)						
**Total (95% CI)**		**236**		**436**	**100.0%**	**1.58 [0.67, 3.72]**
Total events	89		87			
Heterogeneity: Tau² = 0.78; Chi² = 18.63, df = 6 (P = 0.005); I² = 68%						
Test for overall effect: Z = 1.05 (P = 0.29)						
Test for subgroup differences: Chi² = 3.69, df = 1 (P = 0.05), I² = 72.9%						

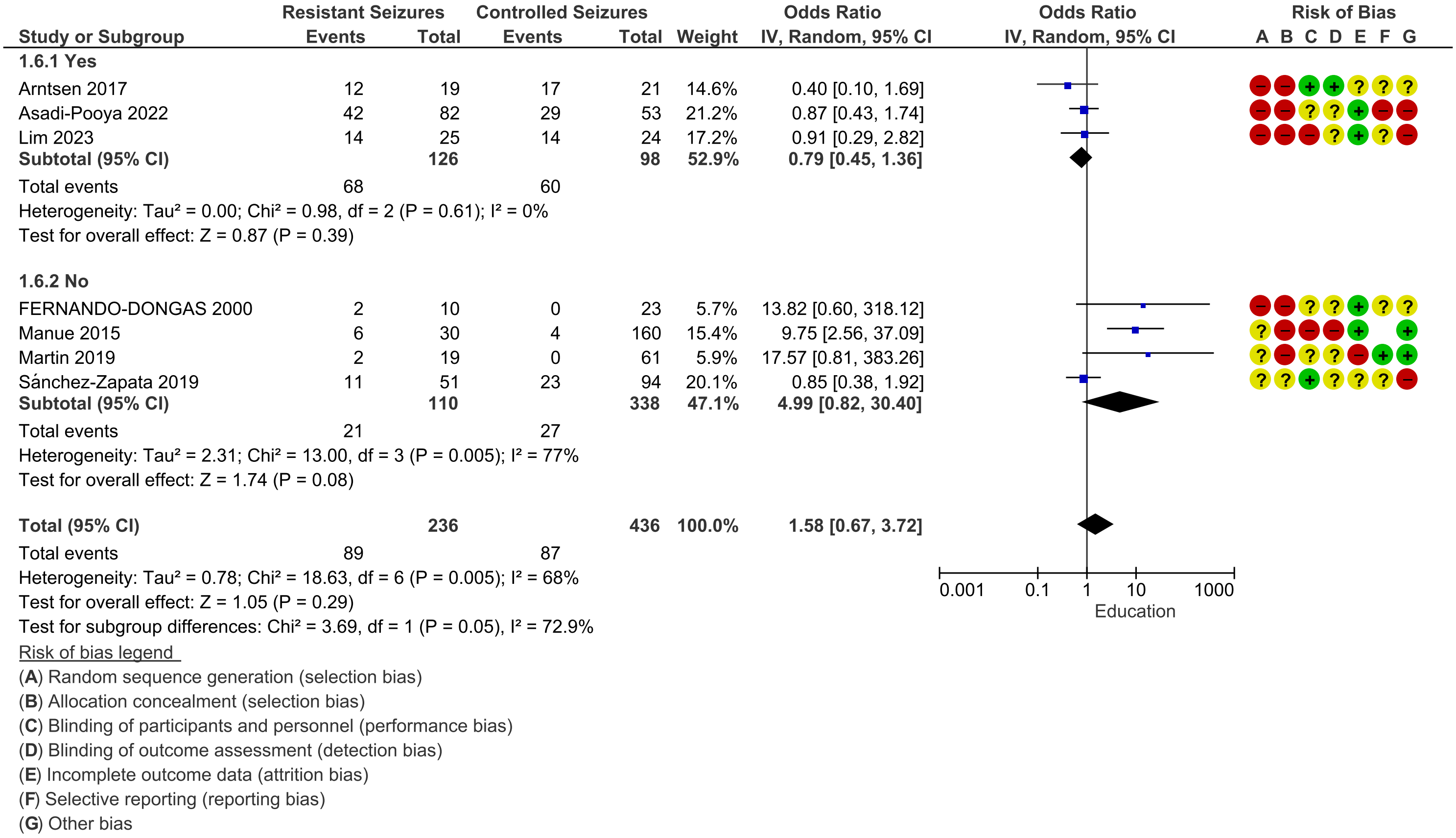

Risk of bias legend
(**A**) Random sequence generation (selection bias)
(**B**) Allocation concealment (selection bias)
(**C**) Blinding of participants and personnel (performance bias)
(**D**) Blinding of outcome assessment (detection bias)
(**E**) Incomplete outcome data (attrition bias)
(**F**) Selective reporting (reporting bias)
(**G**) Other bias

	Resistant Seizures		Controlled Seizures			Odds Ratio	Risk of Bias						
**Study or Subgroup**	**Events**	**Total**	**Events**	**Total**	**Weight**	**IV, Fixed, 95% CI**	**A**	**B**	**C**	**D**	**E**	**F**	**G**
Asadi-Pooya 2014	22	48	23	68	8.8%	1.66 [0.78, 3.53]	−	−		?	+	+	−
Asadi-Pooya 2022	31	82	20	53	10.0%	1.00 [0.49, 2.05]	−	−	?	?	+	−	−
ASLAN 2005	8	20	4	12	2.3%	1.33 [0.30, 5.96]	−	−		?		+	−
AYKUTLU 2004	2	7	27	81	1.8%	0.80 [0.15, 4.40]	−	−	?	?	+	+	?
Cação 2018	33	121	34	119	16.0%	0.94 [0.53, 1.65]	−	−	?	?	?	+	+
Chen 2020	2	23	3	40	1.5%	1.17 [0.18, 7.60]	−	−	?	?	+	+	+
FERNANDO-DONGAS 2000	3	10	7	23	1.9%	0.98 [0.19, 4.94]	−	−	?	?	+	?	?
Gürer 2019	45	83	67	132	16.8%	1.15 [0.66, 1.99]	−	−	?	?	+	+	+
Hernández-Vanegas 2016	31	57	25	46	8.4%	1.00 [0.46, 2.18]	−	−	−	−	−	?	?
Hirano 2008	0	1	12	33	0.5%	0.57 [0.02, 15.17]	−	−	−	?	?	+	−
Jayalakshmi 2014	11	38	63	163	8.6%	0.65 [0.30, 1.39]	−	−	−	−	?	?	−
Lim 2023	3	25	4	24	1.9%	0.68 [0.14, 3.43]	−	−	−	?	+	?	−
Manue 2015	6	30	6	160	3.5%	6.42 [1.91, 21.53]	?	−	−	−	+		+
Sager 2022	8	21	10	41	4.0%	1.91 [0.61, 5.92]	?	−	−	−	+	?	−
Sánchez-Zapata 2019	24	51	46	94	10.9%	0.93 [0.47, 1.84]	?	?	+	?	?	?	−
Senf 2013	1	27	4	39	1.0%	0.34 [0.04, 3.19]	−	−	−	−	+	+	?
Viswanathan 2021	3	22	6	34	2.2%	0.74 [0.16, 3.31]	−	−	−	?	−	?	−
**Total (95% CI)**		**666**		**1162**	**100.0%**	**1.08 [0.86, 1.36]**							
Total events	233		361										

Heterogeneity: Chi² = 14.73, df = 16 (P = 0.54); I² = 0%

Test for overall effect: Z = 0.69 (P = 0.49)

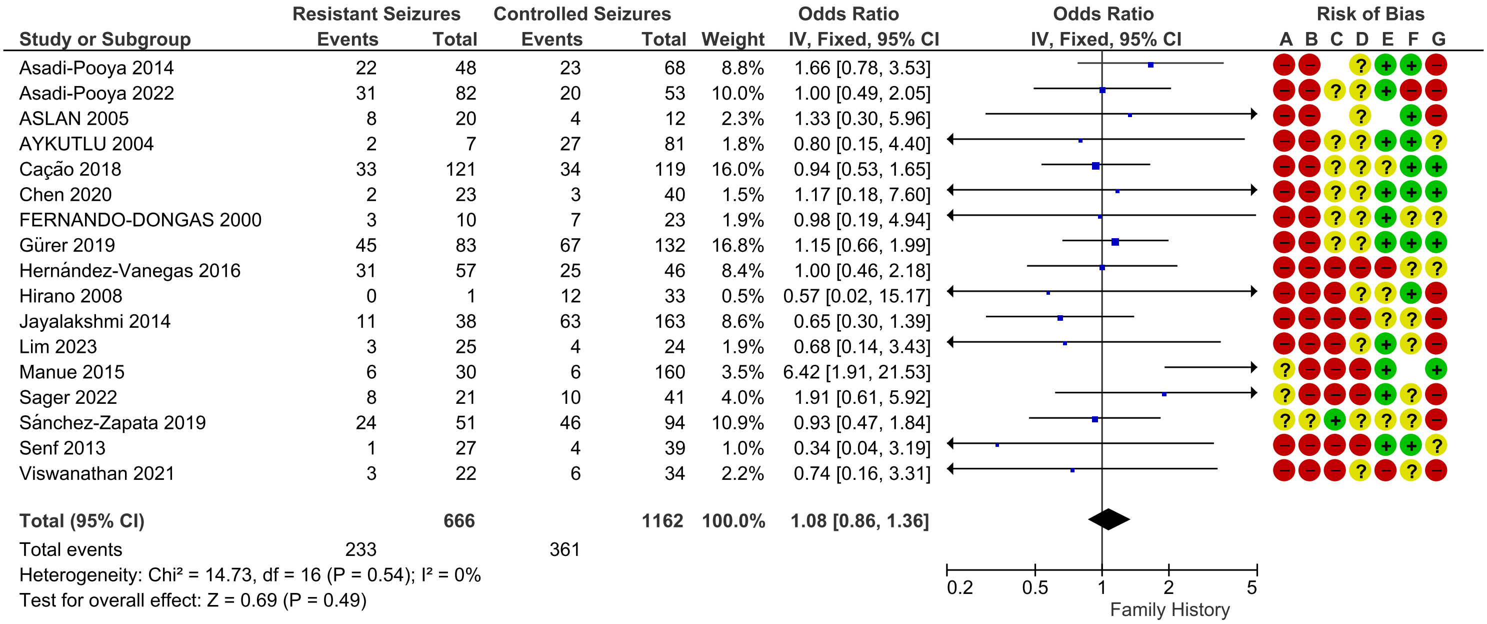

Risk of bias legend

(**A**) Random sequence generation (selection bias)
(**B**) Allocation concealment (selection bias)
(**C**) Blinding of participants and personnel (performance bias)
(**D**) Blinding of outcome assessment (detection bias)
(**E**) Incomplete outcome data (attrition bias)
(**F**) Selective reporting (reporting bias)
(**G**) Other bias
