# Supplementary material for: A systematic review and meta-analysis of factors related to first line drugs refractoriness in patients with juvenile myoclonic epilepsy (JME)
Source: PLoS One. 2024 Apr 9;19(4):e0300930. doi: 10.1371/journal.pone.0300930 (PMC11003615; doi:10.1371/journal.pone.0300930)
Supplement: S1 Table — The search was performed on 22 September 2023 and yielded 792 hits. Publications were filtered on the publication type “Article”. (PDF) [file pone.0300930.s004.pdf]

S1 Table PubMed/MEDLINE search string. *The search was performed on 22 September 2023 and yielded 792 hits. Publications were filtered on the publication type "Article".*

Database	Search String
PubMed/MEDLINE	"Myoclonic Epilepsy, Juvenile"[MeSH] OR "juvenile myoclonic epilepsy"[tiab] OR "Janz syndrome"[tiab] OR JME[tiab] OR "Impulsive Petit Mal"[tiab] AND "Drug Resistance"[MeSH] OR "Prognosis"[MeSH] OR refractory[tiab] OR drug-resistant[tiab] OR "drug resistant"[tiab] OR "seizure outcome"[tiab] OR "seizure control"[tiab] OR "seizure remission"[tiab] OR "seizure free"[tiab] OR seizure-free[tiab] OR pharmacoresistan*[tiab] OR prognosis[tiab]
