# Supplementary material for: A systematic review and meta-analysis of factors related to first line drugs refractoriness in patients with juvenile myoclonic epilepsy (JME)
Source: PLoS One. 2024 Apr 9;19(4):e0300930. doi: 10.1371/journal.pone.0300930 (PMC11003615; doi:10.1371/journal.pone.0300930)
Supplement: S2 Table — The search was performed on 22 September 2023 and yielded 500 hits. Publications were filtered on the publication type “Article”. (PDF) [file pone.0300930.s005.pdf]

S2 Table Scopus search string. *The search was performed on 22 September 2023 and yielded 500 hits. Publications were filtered on the publication type "Article".*

**Database**	**Search String**
Scopus	"Myoclonic Epilepsy, Juvenile" OR "juvenile myoclonic epilepsy" OR "Janz syndrome" OR JME OR "Impulsive Petit Mal" AND "Drug Resistance" OR "Prognosis" OR refractory OR "drug resistant" OR "seizure outcome" OR "seizure control" OR "seizure remission" OR "seizure free" OR seizure-free OR pharmacoresistan* OR prognosis
