# Supplementary material for: A systematic review and meta-analysis of factors related to first line drugs refractoriness in patients with juvenile myoclonic epilepsy (JME)
Source: PLoS One. 2024 Apr 9;19(4):e0300930. doi: 10.1371/journal.pone.0300930 (PMC11003615; doi:10.1371/journal.pone.0300930)
Supplement: S4 Table — Studies can be attributed a maximum of one star (*) for each item. The total score is calculated as the sum of stars. A higher score indicates a better quality of the study. (PDF) [file pone.0300930.s007.pdf]

**Study**	**Selection**				**Compatibility**		**Outcome**			**Score**
	Representativeness of the Exposed Cohort	Selection of the Non - Exposed Cohort	Ascertainment of Exposure	Outcome of Interest Was Not Present at Start of Study	Controls for the Most Important Factor (refractory JME)	Controls for a Second Important Factor	Assessment of Outcome	Follow-Up Long Enough for Outcomes to Occur (>5 years)	Adequacy of Follow Up of Cohorts	
Yam 2017	*	*	*	*	*		*			6
Asadi-Pooya 2022	*	*	*	*			*			5
Lim 2023	*	*	*	*			*			5
Hirano 2008	*	*		*			*			4
Martin 2019	*	*	*	*			*			5
Sanchez-Zapata 2019	*	*	*	*			*			5
Shakeshaft 2022	*	*	*	*	*		*			6
Aykutlu 2005	*	*	*	*	*		*			6
Hofler 2014	*	*						*	*	4
Arntsen 2017	*	*							*	3
Aslan 2005	*	*	*	*			*			5
Guaranha 2010	*	*	*				*	*		5
Senf 2013	*	*					*	*	*	5
Asadi-Pooya 2014	*	*							*	3
Martinovic 2001	*	*	*				*			4
Fernando-Dongas 2000	*	*								2
Gelisse 2001	*	*	*		*			*		5
Manuel 2011	*	*		*			*	*		5
Sager 2021	*	*	*	*	*		*			6
Jayalakshmi 2011	*	*	*		*	*		*	*	7
Vanegas 2012	*	*	*		*		*			5
Cacao 2018	*	*	*		*		*			5
Viswanathan 2018	*	*		*			*			4
Gurer 2019	*	*	*				*			4
Chen 2013	*	*		*			*	*		5

S4 Table. Risk of bias assessment using the Newcastle –Ottawa quality assessment scale for cohort studies. Studies can be attributed a maximum of one star (*) for each item. The total score is calculated as the sum of stars. A higher score indicates a better quality of the study.
