# Supplementary material for: A systematic review and meta-analysis of factors related to first line drugs refractoriness in patients with juvenile myoclonic epilepsy (JME)
Source: PLoS One. 2024 Apr 9;19(4):e0300930. doi: 10.1371/journal.pone.0300930 (PMC11003615; doi:10.1371/journal.pone.0300930)
Supplement: S5 Table — A total of 25 studies describing seizure outcome in 3051 individuals with JME were classified based on the number of drug resistant individuals, number of seizure free individuals, YOS: Year of study, sample size, yi: vector with the observed effect sizes or outcomes, vi: vector with the corresponding sampling variances, pi: vector with the (signed) p-values. (PDF) [file pone.0300930.s008.pdf]

Study	Name	DRE	Seizure Free	YOS	Size	pi	yi	vi
**1**	Asadi-Pooya	48	68	2014	116	0.4137931	0.699503	0.00214592
**2**	Asadi-Pooya	82	53	2022	135	0.60740741	0.89284387	0.00184502
**3**	ASLAN	20	12	2005	32	0.625	0.90789109	0.00769231
**4**	AYKUTLU	7	88	2004	95	0.07368421	0.28313393	0.0026178
**5**	Cacao	121	119	2018	240	0.50416667	0.78954762	0.0010395
**6**	Chen	23	40	2020	63	0.36507937	0.65095399	0.00393701
**7**	FERNANDO-DONGAS	10	23	2000	33	0.3030303	0.58912256	0.00746269
**8**	Gurer	83	132	2019	215	0.38604651	0.67097483	0.00116009
**9**	Vanegas	57	46	2016	103	0.55339806	0.83838437	0.00241546
**10**	Hirano	1	46	2008	47	0.0212766	0.17520621	0.00526316
**11**	Jayalakshmi	38	163	2014	201	0.18905473	0.45177569	0.00124069
**12**	Lim	25	24	2023	49	0.51020408	0.79540083	0.00505051
**13**	Manuel	22	22	2015	44	0.5	0.78539816	0.00561798
**14**	Sager	21	41	2022	62	0.33870968	0.62384968	0.004
**15**	Sanchez-Zapata	51	94	2019	145	0.35172414	0.63591732	0.00171821
**16**	Senf	27	39	2013	66	0.40909091	0.69534962	0.0037594
**17**	Viswanathan	22	34	2021	56	0.39285714	0.67932416	0.00442478
**18**	Gelisse	24	116	2001	140	0.17142857	0.42995158	0.00177936
**19**	Martin	26	61	2019	87	0.29885057	0.58086232	0.00285714
**20**	Guaranha	40	25	2011	65	0.61538462	0.90005039	0.00381679
**21**	MARTINOVICÂ´	22	36	2001	58	0.37931034	0.66559292	0.0042735
**22**	Shakeshaft	165	600	2022	765	0.21568627	0.48343064	0.00032658
**23**	Yam	8	11	2022	19	0.42105263	0.71001683	0.01282051
**24**	Arntsen	19	21	2017	40	0.475	0.76099095	0.00617284
**25**	Hofler	66	109	2014	175	0.37714286	0.66198775	0.0014245

S5 Table. **Details of all 25 included studies.** A total of 25 studies describing seizure outcome in 3051 individuals with JME were classified based on the number of drug resistant individuals, number of seizure free individuals, YOS: Year of study, sample size, yi: vector with the observed effect sizes or outcomes, vi: vector with the corresponding sampling variances, pi: vector with the (signed) p-values.
