# Supplementary material for: A systematic review and meta-analysis of factors related to first line drugs refractoriness in patients with juvenile myoclonic epilepsy (JME)
Source: PLoS One. 2024 Apr 9;19(4):e0300930. doi: 10.1371/journal.pone.0300930 (PMC11003615; doi:10.1371/journal.pone.0300930)
Supplement: Attachment — Submitted filename: Response to Reviewers.pdf [file pone.0300930.s010.pdf]

Dear All,

I appreciate the thorough review conducted by the academic editor and the reviewers for my manuscript submission to PLOS ONE. I am pleased to receive constructive feedback and guidance on how to enhance the quality of the manuscript.

I have carefully considered the reviewers' comments and suggestions, and I am committed to addressing them in the revised version of the manuscript. I am thankful for the positive assessment provided by Reviewer #2 and have taken note of the specific recommendations outlined in their comments. I have ensured that these suggestions are incorporated into the revised manuscript. For Reviewer #1's comments, particularly regarding the risk of bias assessment tools, I acknowledge the oversight in using Cochrane's ROB instead of the Newcastle-Ottawa Scale (NOS) for non-randomized studies. I appreciate the guidance provided, and I will address this issue.

You can find my answers to your comments as following:

Concerning ➔ Reviewer #1:

1 - Although the authors mentioned about Newcastle-Ottawa scale (NOS) for risk of bias assessments, they used Cochrane's ROB and did not use NOS. It is not appropriate. Please

I completely understand your concern, and I have addressed the issue since your last comment. I have now differentiated between the studies included in the paper, categorizing them as either randomized or non-randomized/quasi-randomized studies. Subsequently, I reapplied the Cochrane Risk of Bias (COB) tool to the studies, evaluating the margin of bias. Recognizing the limitations of COB in determining bias for non-randomized studies, I proceeded to supplement it with the Newcastle-Ottawa Scale (NOS) for a more comprehensive assessment.

To provide clarity on this methodology, I have included an explanation in the revised manuscript from line 260 to line 269.

"In response to these findings, we incorporated the Newcastle–Ottawa quality assessment scale - NOS - into our methodology to provide a nuanced evaluation for these studies [74]. The detailed assessment can be accessed in the supplementary S4 table. The Newcastle–Ottawa quality assessment scale was systematically applied to assess the methodological quality of the studies, considering three major components: cohort selection, comparability, and assessment of outcome. The scale operates on a scoring system ranging from 0 to 9, with studies considered to be of high quality if they score ≥ 5 and of low quality if they score < 5. This additional layer of evaluation was deemed necessary to ensure a comprehensive and accurate assessment of potential biases, particularly in the context of non-randomized control studies."

Furthermore, I have introduced Table S4 in the supplementary section to enhance the presentation of relevant information.

Study	Selection				Compatibility		Outcome			Score
	Representativeness of the Exposed Cohort	Selection of the Non - Exposed Cohort	Ascertainment of Exposure	Outcome of Interest Was Not Present at Start of Study	Controls for the Most Important Factor (refractory JME)	Controls for a Second Important Factor	Assessment of Outcome	Follow-Up Long Enough for Outcomes to Occur (>5 years)	Adequacy of Follow Up of Cohorts	
Yam 2017	*	*	*	*	*		*			6
Asadi-Pooya 2022	*	*	*	*			*			5
Lim 2023	*	*	*	*			*			5
Hirano 2008	*	*		*			*			4
Martin 2019	*	*	*	*			*			5
Sanchez-Zapata 2019	*	*	*	*			*			5
Shakeshaft 2022	*	*	*	*	*		*			6
Aykutlu 2005	*	*	*	*	*		*			6
Hofler 2014	*	*						*	*	4
Arntsen 2017	*	*							*	3
Aslan 2005	*	*	*	*			*			5
Guaranha 2010	*	*	*				*	*		5
Senf 2013	*	*					*	*	*	5
Asadi-Pooya 2014	*	*							*	3
Martinovic 2001	*	*	*				*			4
Fernando-Dongas 2000	*	*								2
Gelisse 2001	*	*	*		*			*		5
Manuel 2011	*	*		*			*	*		5
Sager 2021	*	*	*	*	*		*			6
Jayalakshmi 2011	*	*	*		*	*		*	*	7
Vanegas 2012	*	*	*		*		*			5
Cacao 2018	*	*	*		*		*			5
Viswanathan 2018	*	*		*			*			4
Gurer 2019	*	*	*				*			4
Chen 2013	*	*		*			*	*		5

S4 Table. Risk of bias assessment using the Newcastle –Ottawa quality assessment scale for cohort studies. Studies can be attributed a maximum of one star (*) for each item. The total score is calculated as the sum of stars. A higher score indicates a better quality of the study.

Thank you for bringing this to my attention, and I appreciate your diligence in reviewing the manuscript. If you have any further suggestions or concerns, please feel free to let me know.

Concerning ➔ Reviewer #2:

1 - Line 38

While the term "95%CI" is mentioned in the abstract, the actual range of 95% confidence intervals for each odds ratio is not provided. Additionally, for clarity, it is recommended to spell out "OR" and "CI" when they first appear in the abstract.

I have ensured to fix and include this information for a more comprehensive presentation of the results.

2 - Line 70

The authors introduced the term "idiopathic generalized epilepsy" at line 70. Considering the apparent similarity between GGE and IGE within the manuscript's context, it is advisable to consistently use one of these terms throughout.

Well received. I have changed it to prevent using 2 terms. I will adhere to GGE in the manuscript based on your preferences.

3 - Line 72

GGE is spelled out repeatedly. GGE is already defined previously.

It's corrected through the manuscript.

4 - Line 93,137 and 369

JME is spelled out repeatedly.

Well received and corrected.

5 - Line 188

There may be a typographical error, and "seizure-resistant" could potentially be corrected to "drug-resistant" to better align with the natural context.

Well received and corrected.

6 - Line 199

It is suggested to maintain consistency by adhering to the abbreviation "VPA" for valproic acid, as previously defined.

It's corrected based on your comment.

7 - Figure 6 is interesting. It seems that the regression curve is showing upward trend. Is there possibility that the prevalence of DRE among JME is increasing over the years?

I appreciate the insightful observation made by the reviewer regarding Figure 6. I would like to provide a detailed explanation for the apparent upward trend in the regression curve, suggesting a potential increase in the prevalence of drug-resistant Juvenile Myoclonic Epilepsy (JME) patients over the years:

After meticulous analysis and considering the available literature, it is indeed accurate to observe a slight increase in the number of drug-resistant JME patients post the year 2010. This increase can be primarily attributed to the significant characterization that occurred in 2010 when the International League Against Epilepsy (ILAE) introduced a more refined definition for drug resistance in JME. Consequently, a better-defined criterion for both JME and Drug-Resistant Epilepsy (DRE) led to improved diagnosis and characterization.

The enhancement in diagnosis and characterization has been progressively evolving from 2010 onwards, contributing to a more accurate identification of drug-resistant cases. However, it is crucial to note that the treatment options, notably Valproic Acid (VPA), remain consistent throughout this period.

In addition to the refinement in characterization, other factors, such as genetic considerations and the inheritance of JME, may also play a role in the observed increase. In my thesis I have provided a comprehensive discussion on the complex genetic inheritance of JME, with various genetic loci highlighted. While these factors might contribute to the rise, it is essential to acknowledge the need for further research to fully understand and validate these potential influences.

8 - In table 4, P values should be described as they are instead of “<0.05”. They can be written as like “<0.001” only when they are much smaller than 0.05.

Well received and corrected.

Concerning the journal guidelines for the figures:

All the figures were rechecked using the digital diagnostic tool provided, https://pacev2.apexcovantage.com/.

Concerning the references:

We have added references to the revised manuscript as some sections were added according to the preferences of the reviewers, kindly find the citations and the number of the references added below:

References

[4] Yacubian EM. Juvenile myoclonic epilepsy: Challenges on its 60th anniversary. Seizure. 2017;44:48–52. doi:10.1016/j.seizure.2016.09.005.

[8] Iqbal N, Caswell H, Muir R, Cadden A, Ferguson S, Mackenzie H, Watson P, Duncan S. Neuropsychological profiles of patients with juvenile myoclonic epilepsy and their siblings: An extended study. Epilepsia. 2015 Aug;56(8):1301-8. doi:https://doi.org/10.1111/epi.13061.

[20] Yam M, Glatt S, Nosatzki S, Mirelman A, Hausdorff JM, Goldstein L, et al. Limited Ability to Adjust N2 Amplitude During Dual Task Walking in People With Drug-Resistant Juvenile Myoclonic Epilepsy. Frontiers in Neurology. 2022;13:793212. doi:10.3389/fneur.2022.793212.

[21] Asadi-Pooya AA, Rostamihosseinkhani M, Farazdaghi M. Seizure and social outcomes in patients with juvenile myoclonic epilepsy (JME). Seizure. 2022;97:15–19. doi:10.1016/j.seizure.2022.03.002.

[23] Hirano Y, Oguni H, Osawa M. Clinical factors related to treatment resistance in juvenile myoclonic epilepsy. Rinsho Shinkeigaku. 2008;48(10):727–732. doi:10.5692/clinicalneurol.48.727.

[24] Sanchez Zapata P, Zapata Berruecos JF, Pel ˜ A¡ez Sanchez RG, Molina Castao CF. Seizure control with valproic acid, lamotrigine or levetiracetam in the management of drug-resistant juvenile myoclonic epilepsy. Systematic review and meta-analysis. Neurologia Argentina. 2022;14(1):26–36. doi:10.1016/j.neuarg.2021.08.003.

[26] Aykutlu, E., Baykan, B., Baral-Kulaksızoglu, I., Gurses, C., Gokyigit, A. Clinical and EEG Features of Therapy-Resistant Patients with Juvenile Myoclonic Epilepsy. Epilepsi J Turk Epilepsy Soc. 2004;10(2), 100-105.

[27] Hofler J, Unterberger I, Dobesberger J, Kuchukhidze G, Walser G, Trinka E. Seizure outcome in 175 patients with juvenile myoclonic epilepsy - A long-term observational study.

Epilepsy Research. 2014;108(10):1817–1824. doi:10.1016/j.eplepsyres.2014.09.008. February 23, 2024 26/33

[28] Aslan K, Bozdemir H, Bicakci S¸, Sarica Y, Sertdemir Y. Role of the Risk Factors and Family History on the Prognosis of Patients with Juvenile Myoclonic Epilepsy. Arch Epilepsy 2005;11:70-76.

[29] Arntsen V, Sand T, Syvertsen MR, Brodtkorb E. Prolonged epileptiform EEG runs are associated with persistent seizures in juvenile myoclonic epilepsy. Epilepsy Research. 2017;134:26–32. doi:10.1016/j.eplepsyres.2017.05.003.

[32] Asadi-Pooya AA, Hashemzehi Z, Emami M. Predictors of seizure control in patients with juvenile myoclonic epilepsy (JME). Seizure. 2014;23(10):889–891. doi:10.1016/j.seizure.2014.08.004.

[33] Martinovic; ˚A. Adjunctive behavioural treatment in adolescents and young adults with juvenile myoclonic epilepsy. Seizure. 2001;10(1):42–47. doi:10.1053/seiz.2000.0479.

[34] Fernando-Dongas MC, Radtke RA, VanLandingham KE, Husain AM. Characteristics of valproic acid resistant juvenile myoclonic epilepsy. Seizure. 2000;9(6):385–388. doi:10.1053/seiz.2000.0432.

[36] Manuel D, C B R, Alexander A. CLINICAL, NEUROPSYCHOLOGICAL AND NEUROPHYSIOLOGICAL CORRELATES OF DRUG RESISTANT JUVENILE MYOCLONIC EPILEPSY. Journal of Evidence Based Medicine and Healthcare. 2015;2(36):5658–5668. doi:10.18410/jebmh/2015/780. February 23, 2024 27/33

[37] Sager S, Cag Y, Akin Y. Determination of the factors that cause valproic acid resistance in the pediatric juvenile myoclonic epilepsy cohort. Medicine Science | International Medical Journal. 2022;11(1):338. doi:10.5455/medscience.2021.08.244.

[38] Jayalakshmi S, Vooturi S, Bana AK, Sailaja S, Somayajula S, Mohandas S. Factors associated with lack of response to valproic acid monotherapy in juvenile myoclonic epilepsy. Seizure. 2014;23(7):527–532. doi:10.1016/j.seizure.2014.03.017.

[39] Hernandez-Vanegas LE, Jara-Prado A, Ochoa A, Rodraguez Y Rodraguez N, Duran RM, Crail-Melandez D, et al. High-dose versus low-dose valproate for the treatment of juvenile myoclonic epilepsy: Going from low to high. Epilepsy & Behavior. 2016;61:34–40. doi:10.1016/j.yebeh.2016.04.047.

[40] Cacao G, Parra J, Mannan S, Sisodiya SM, Sander JW. Juvenile myoclonic epilepsy refractory to treatment in a tertiary referral center. Epilepsy and Behavior. 2018;82:81–86. doi:10.1016/j.yebeh.2018.03.002.

[41] Viswanathan LG, Mundlamuri RC, Raghavendra K, Asranna A, Chatterjee A, Gautham B, et al. Long-Term Seizures Outcome in Juvenile Myoclonic Epilepsy (JME): A Retrospective Cohort Study in an Indian Population. International Journal of Epilepsy. 2021;7(01):15–21. doi:10.1055/s-0041-1729459.

[43] Chen C, Lee H, Chen C, Kwan S, Chen S, . Short-term results of vagus nerve stimulation in pediatric patients with refractory epilepsy. Pediatrics & ˆa€¦. 2012;(Query date: 2023-09-22 11:16:22).

[72] Mourad Ouzzani, Hossam Hammady, Zbys Fedorowicz, and Ahmed Elmagarmid. Rayyan — a web and mobile app for systematic reviews. Systematic Reviews (2016) 5:210 doi:10.1186/s13643-016-0384-4.

[73] Viechtbauer W. Conducting meta-analyses in R with the metafor package. Journal of Statistical Software. 2010;36(3), 1–48. doi:10.18637/jss.v036.i03.

[74] Lo, C.KL., Mertz, D. and Loeb, M. Newcastle-Ottawa Scale: comparing reviewers' to authors' assessments. BMC Med Res Methodol 14, 45 (2014). doi:10.1186/1471-2288-14-45.

I would also like to express my gratitude for the positive feedback on the clarity and language of the manuscript from both reviewers. I have carefully proofreaded the revised version to address any remaining typographical or grammatical errors.

Thank you for the opportunity to improve and resubmit my manuscript to PLOS ONE. I look forward to your feedback on the revised version.

Kind regards,
